# Hepatic and pulmonary macrophage activity in a mucosal challenge model of Ebola virus disease

**DOI:** 10.3389/fimmu.2024.1439971

**Published:** 2024-11-20

**Authors:** Timothy G. Wanninger, Omar A. Saldarriaga, Esteban Arroyave, Daniel E. Millian, Jason E. Comer, Slobodan Paessler, Heather L. Stevenson

**Affiliations:** ^1^ Department of Pathology, University of Texas Medical Branch, Galveston, TX, United States; ^2^ Department of Microbiology and Immunology, University of Texas Medical Branch, Galveston, TX, United States; ^3^ Department of Microbiology and Immunology, Louisiana State University Health Shreveport, Shreveport, LA, United States

**Keywords:** Ebola, macaque, macrophage, liver, lung, CD163, IDO1, MAC387

## Abstract

**Background:**

The inflammatory macrophage response contributes to severe Ebola virus disease, with liver and lung injury in humans.

**Objective:**

We sought to further define the activation status of hepatic and pulmonary macrophage populations in Ebola virus disease.

**Methods:**

We compared liver and lung tissue from terminal Ebola virus (EBOV)-infected and uninfected control cynomolgus macaques challenged via the conjunctival route. Gene and protein expression was quantified using the nCounter and GeoMx Digital Spatial Profiling platforms. Macrophage phenotypes were further quantified by digital pathology analysis.

**Results:**

Hepatic macrophages in the EBOV-infected group demonstrated a mixed inflammatory/non-inflammatory profile, with upregulation of CD163 protein expression, associated with macrophage activation syndrome. Hepatic macrophages also showed differential expression of gene sets related to monocyte/macrophage differentiation, antigen presentation, and T cell activation, which were associated with decreased MHC-II allele expression. Moreover, hepatic macrophages had enriched expression of genes and proteins targetable with known immunomodulatory therapeutics, including S100A9, IDO1, and CTLA-4. No statistically significant differences in M1/M2 gene expression were observed in hepatic macrophages compared to controls. The significant changes that occurred in both the liver and lung were more pronounced in the liver.

**Conclusion:**

These data demonstrate that hepatic macrophages in terminal conjunctivally challenged cynomolgus macaques may express a unique inflammatory profile compared to other macaque models and that macrophage-related pharmacologically druggable targets are expressed in both the liver and the lung in Ebola virus disease.

## Introduction

Ebola virus disease (EVD) is a systemic illness associated with a dysregulated inflammatory response with severe, and often fatal outcomes. Five virus species from the genus *Orthoebolavirus*, *O. zairense, O. sudanense, O. bundibugyoense, O. taiense*, and *O. restonense*, have all caused infections in humans (1). Signs and symptoms of Ebola virus disease include fever, myalgia, headache, nausea, vomiting, diarrhea, rash, and hemorrhage (2). The largest outbreak to date occurred in West Africa and resulted in over 28,000 cases and 11,325 deaths (3). Historical case fatality rates have ranged widely, from 25% to 91%, depending on the specific outbreak (1).

In response to this disease, multiple monoclonal antibody therapies and vaccines have been developed to prevent infection and treat disease. Monoclonal therapies include antibodies produced to inhibit virus entry into cells (4, 5). Currently, only two antibody therapies are FDA-approved. In addition, there are two current vaccines, Ervebo (rVSV∆G-ZEBOV-GP, live, attenuated), approved by the United States Food and Drug Administration and the European Medicines Agency, and Zabdeno (Ad26.ZEBOV-GP [recombinant])/Mvabea (MVA-BN-Filo [recombinant]), a vaccine combination series approved by the European Medicines Agency, as well as multiple other candidate vaccines at various stages of development or approval for use for infection prevention (6–9). However, even with treatment, outcomes are poorer in cases with high viral loads or those who present late to care (10).

Macrophages are key contributors to the inflammatory immune signaling promoting systemic disease (11). Human monocyte-derived macrophages infected with Ebola virus (EBOV) produce multiple cytokines and chemokines (12). Infection of these cells by EBOV can be affected by the polarization state, which has historically been classified using CD14 and CD16: classical (CD14^+^CD16^-^, M1) and non-classical (CD14^-^CD16^+^, M2) macrophages (13). Macrophage polarization toward a M2a phenotype by a combination of IL-4/IL-13 is associated with EBOV glycoprotein-mediated entry into human monocyte-derived macrophages by EBOV GP-expressing recombinant vesicular stomatitis virus (14, 15). Similarly, exposure to IL-10, which is associated with M2c polarization, enhances EBOV virus-like particle entry into human monocyte-derived macrophages (14, 16). Conversely, IFN-γ, which is associated with M1 polarization, reduces murine peritoneal macrophage infection by EBOV (14, 17). In EBOV-infected rhesus macaques, hepatic CD14 staining temporally increases, while CD16 staining decreases, consistent with the accumulation of classical macrophages over time (18).

The liver, an organ populated by macrophages, is one of the susceptible sites for orthoebolavirus infection (19, 20). Two major populations of macrophages are relevant in the liver: resident macrophages, called Kupffer cells, and monocyte-derived macrophages, recruited from the systemic circulation, especially following tissue injury (13). Both Kupffer cells and monocyte-derived macrophages can be infected by orthoebolaviruses (11). C-type lectins, including DC-SIGN and DC-SIGNR may facilitate EBOV binding to macrophages (11, 21). LSECtin, another C-type lectin, is expressed by Kupffer cells (22, 23). Increased C-type lectin expression is associated with M2a macrophage polarization in an EBOV GP-expressing recombinant vesicular stomatitis virus mouse model (15). EBOV antigen has been reported to be associated with both CD68^+^ (a Kupffer cell marker) and CD163^+^ (a restorative macrophage marker, including M2 macrophages) macrophages in the liver (13, 24, 25). Soluble CD163, a marker associated with macrophage activation syndrome, is elevated in some patients with Ebola virus disease (25). At a systemic level, hepatic inflammation can result in the release cytokines and other immune mediators into the circulation and shape inflammatory responses throughout the body (26). Thus, understanding the activation status of hepatic macrophages will not only shed light on potential mechanisms of liver injury, but also the overall inflammatory response to orthoebolavirus infection.

Detailed characterization of hepatic macrophages within their tissue context may be accomplished using novel technologies quantifying *in situ* multiplex gene and protein expression. To date, the study of hepatic macrophages in Ebola virus disease have often been done via histological description, immunohistochemistry, and *in situ* hybridization. These studies have highlighted the accumulation of macrophages within the liver during infection, the timing of macrophage infection, and macrophage progression to necrosis (11). More recent work has employed multi-color fluorescence microscopy, showing a shift from CD16^+^ to CD14^+^ hepatic macrophages in EBOV-infected rhesus macaques and a correlation between Tissue Factor (CD142) expression by hepatic macrophages with systemic Tissue Factor activity in EBOV-infected cynomolgus macaques (18, 24). While high-plex gene expression studies have been conducted using single-cell RNA sequencing, this approach often requires extraction of the cells from the tissue, removing the cellular context (27, 28). Multiple platforms have been developed for the *in situ* analysis of the transcriptome, proteome, and metabolites (29). Among these platforms, GeoMx Digital Spatial Profiling performs transcriptomic and high-plex protein expression analysis and is compatible with formalin-fixed tissues, which are commonly collected in animal studies with Risk Group 4 pathogens (30).

Primates are a valuable model for orthoebolavirus infection because they replicate important elements of the infection in humans. For example, primates, like humans, present with liver injury, cytokine storm, signs of hemorrhage, and lymphopenia (31). While other animal models are available for the study of ebolavirus disease, these come with important limitations. For example, mouse models are cheap, come in a variety of transgenic and knockout strains, and have many compatible biochemical and immunologic tools. However, these models require the use of mouse-adapted virus strains, precluding the study of wild-type strains that is possible in primates (31). Historically, primate models are challenged with orthoebolaviruses by the intramuscular route. Beyond the intramuscular route, various mucosal challenge routes more closely mimicking natural exposure, including the intranasal, oral, and conjunctival routes, have also been studied (32–37). Furthermore, the conjunctival challenge route in cynomolgus macaques also better replicates the duration of the human disease compared to the intramuscular challenge model (38).

In this study, we sought to describe the activation status of macrophages within the liver during orthoebolavirus infection. We compared tissues from conjunctivally challenged, EBOV-infected cynomolgus macaques to tissues from healthy controls. The expression of immunology-related genes was quantified from whole liver lysate using nCounter Sprint Profiler analysis. Region-specific gene and protein expression within the liver, including expression by macrophages, was performed using GeoMx Digital Spatial Profiling (GeoMx DSP). In addition, we quantified the presence of macrophages within the liver using digital pathology software. Parallel analyses were also performed on tissues from the lung, another macrophage-rich organ.

## Results

We studied liver and lung tissue collected from cynomolgus macaques infected with EBOV and compared these to tissues from uninfected cynomolgus macaques. As previously reported, the EBOV-infected macaques developed systemic viremia and uniformly lethal disease and met criteria for euthanasia at 7-10 days after exposure. Clinical and laboratory findings were consistent with severe EVD, including biomarkers of liver injury and hematologic abnormalities (**Supplementary Figure S1**) (38).

### Liver and lung histopathology

**Figure 1:**
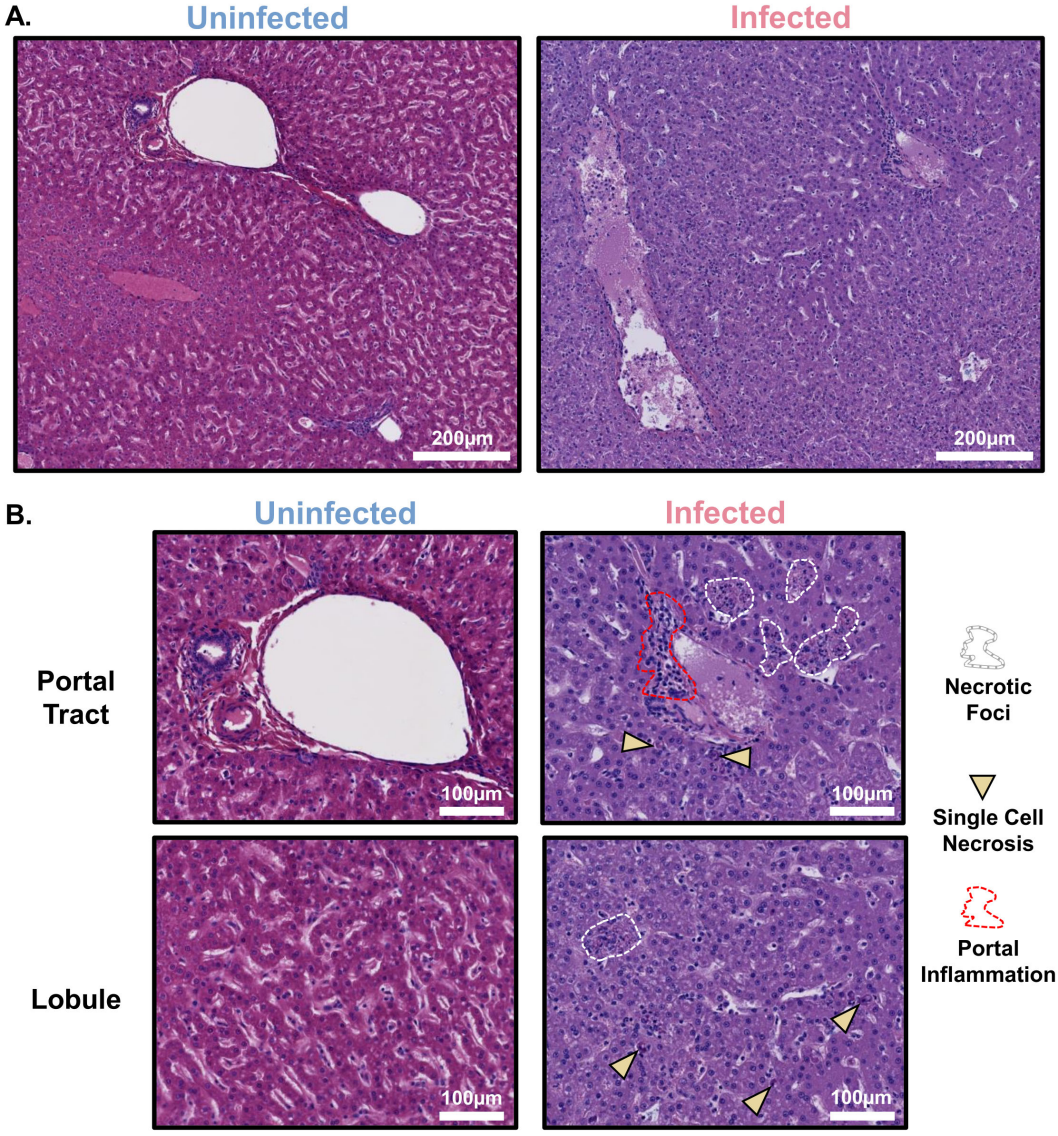
Liver injury in fatal cynomolgus macaque model of EBOV infection includes hepatocellular injury and mononuclear cell accumulation. **(A)** Representative low-magnification images of hematoxylin and eosin-stained liver from uninfected control and EBOV-infected cynomolgus macaques at the time of euthanasia. **(B)** Representative high-magnification images of hematoxylin and eosin-stained liver lobule and portal tract from uninfected control and EBOV-infected cynomolgus macaques at the time of euthanasia. n=5 macaques/group.

We performed new histopathological analyses of the liver and lungs, both macrophage-rich organs, comparing a group of EBOV-infected macaques and a sex-matched set of control macaques to assess the tissue injury present in the infected macaques. Compared to the control macaques, the EBOV-infected macaques showed portal inflammation and necrotic foci (composed of dying hepatocytes, macrophages, and some neutrophils) as well as single necrotic hepatocytes and increased intravascular leukocytes (**Figure 1**). Mononuclear accumulation consistent with interstitial pneumonia was present in the lungs of the EBOV-infected macaques (**Supplementary Figure S2**).

### Macrophage marker quantification in liver and lung

**Figure 2:**
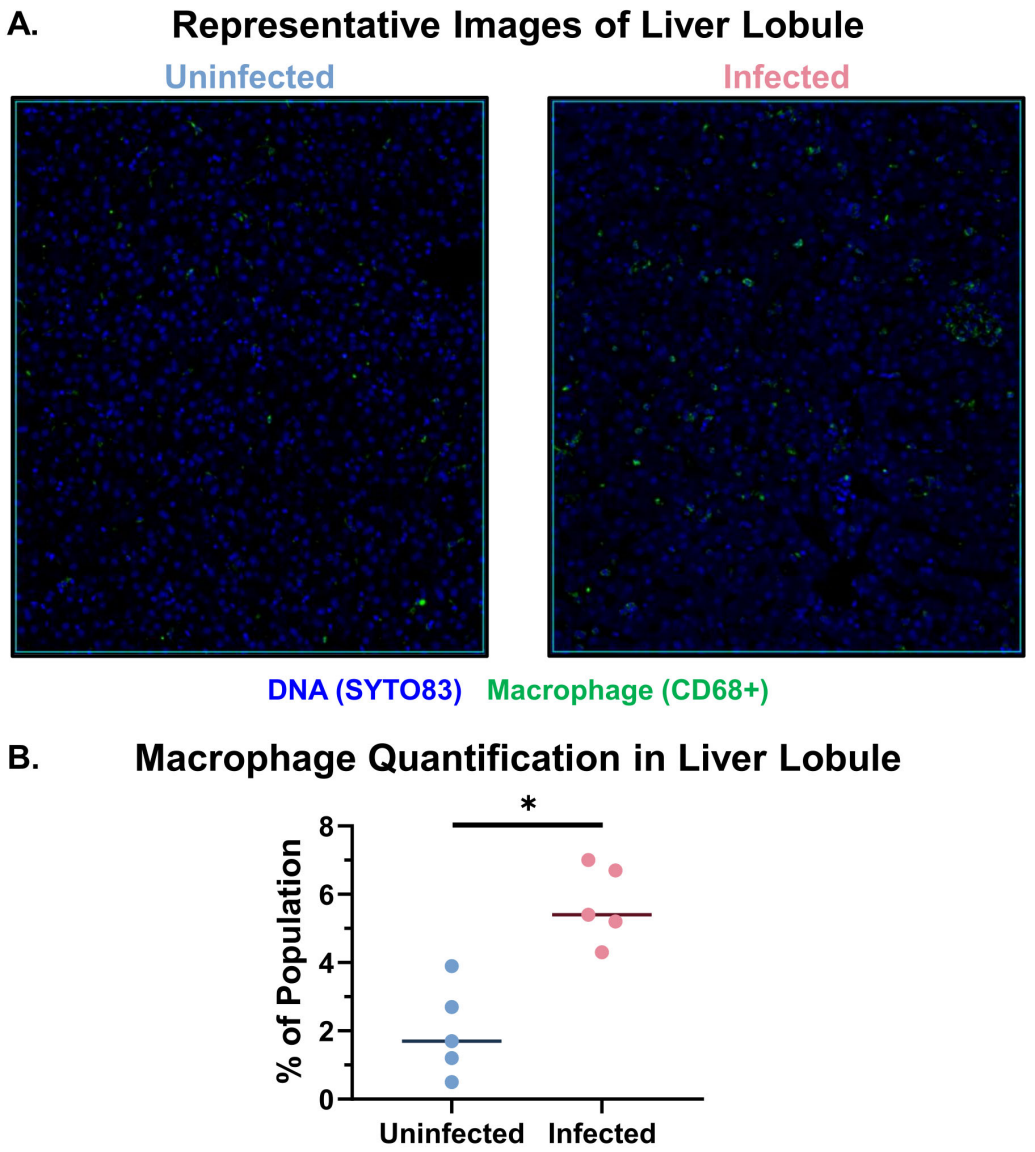
Macrophage accumulation is observed in the livers of EBOV-infected cynomolgus macaques. **(A)** Representative images of the lobular regions of interest analyzed using GeoMx DSP in uninfected and infected macaques. These images show macrophages (CD68^+^ cells, Green), and nuclei (Syto83, Blue). **(B)** Quantification of the percentage of macrophages (CD68^+^ cells) in the lobular region of interest populations. Mann-Whitney test with Benjamini, Krieger, and Yekutieli correction for multiple comparisons (False Discovery Rate 5%) was performed between the groups (EBOV-infected macaques (n=5), uninfected macaques (n=5) (*p<0.05).

In addition to histologically assessing the liver and lung, we also quantified changes in the macrophage population between the uninfected and infected groups. We used Visiopharm digital pathology software to analyze the immunofluorescence images from the GeoMx DSP assay, quantifying the presence of CD68^+^ (macrophage marker) cells (**Figure 2**). Of the total cell population in the liver lobule, the percentage of macrophages was significantly increased (5.4% vs 1.7%, p<0.05) in EBOV-infected versus control macaques, respectively. Of the total cell population in the pulmonary alveoli, the percentage of macrophages was also significantly increased (10.2% vs 0.9%, p<0.05) in EBOV-infected versus control macaques, respectively (**Supplementary Figure S3**). Thus, as is well known, macrophages similarly accumulate in both hepatic lobules and pulmonary alveoli in terminal EBOV-infected macaques.

**Figure 3:**
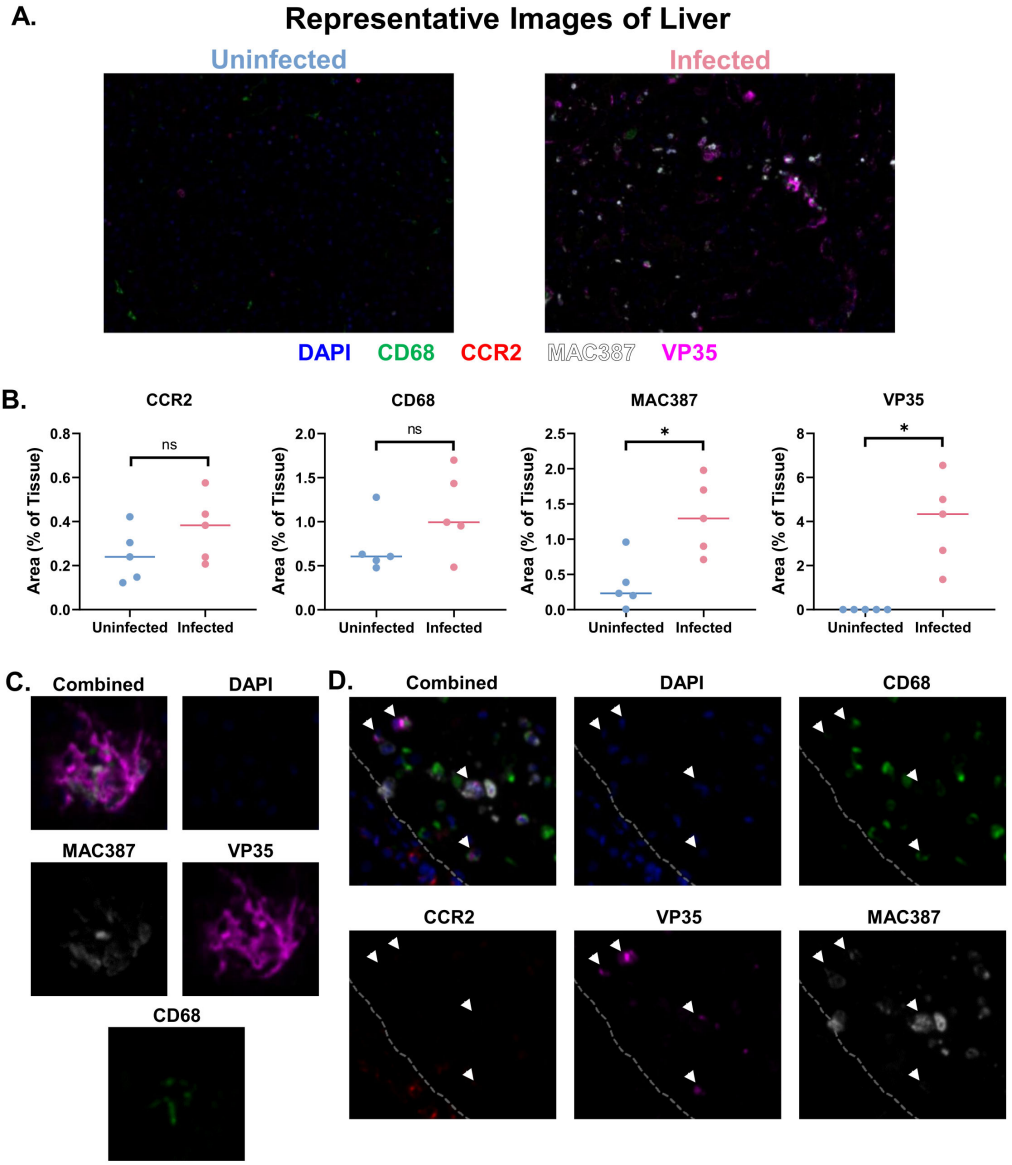
Multiplex spectral imaging microscopy of macrophage populations in the liver. **(A)** Representative Images (20X) of multiplex panel in the liver of uninfected and infected macaques. **(B)** Between-groups comparison of macrophage and EBOV (VP35) markers by positive tissue area. Mann-Whitney Test with Benjamini, Krieger, and Yekutieli correction for multiple comparisons (False Discovery Rate: 5%) was performed (*p<0.05, ns, not significant). **(C)** Representative image (20X) of necrotic foci in EBOV-infected macaques. **(D)** Representative image (20X) of intravascular EBOV antigen-positive cells in EBOV-infected macaques (gray dashed line: intravascular (right), parenchyma (left), white arrows: VP35^+^ cells), n=5 macaques/group.

To further characterize the macrophage populations in the liver and lung, as well as their association with EBOV antigen, we next performed multiplex spectral imaging microscopy. The spectral imaging panel consisted of CD68 (macrophage marker), CCR2 (chemokine receptor associated with macrophage recruitment), MAC387 (recently recruited macrophage marker), and VP35 (EBOV marker). Cell phenotyping with the digital pathology platform, Visiopharm, was performed on the entire tissue, including both the lobular and portal tract regions, with the vascular spaces subtracted. In the liver, VP35 antigen and increased MAC387 staining were detected in EBOV-infected macaques. While CCR2 and CD68 staining showed trends of increased stain area in EBOV-infected macaques, there was no statistically significant difference between the uninfected and infected macaque groups (**Figures 3A, B**). Additionally, we observed the presence of CD68^+^ cells and MAC387^+^ cells in larger areas of VP35 accumulation (**Figure 3C**) as well as intravascular VP35^+^CD68^+^ double-positive and MAC387^+^ cells (**Figure 3D**). We also further characterized the pulmonary macrophage populations in these groups using the same spectral imaging panel. Increases in the CD68^+^, MAC387^+^, and VP35^+^ stain areas were all seen in EBOV-infected macaques as compared to the uninfected group (**Supplementary Figures S4A, B**). VP35^+^CD68^+^ double-positive cells were also observed in the lungs of EBOV-infected macaques (**Supplementary Figure S4C**).

### Whole liver immune-related gene expression

**Figure 4:**
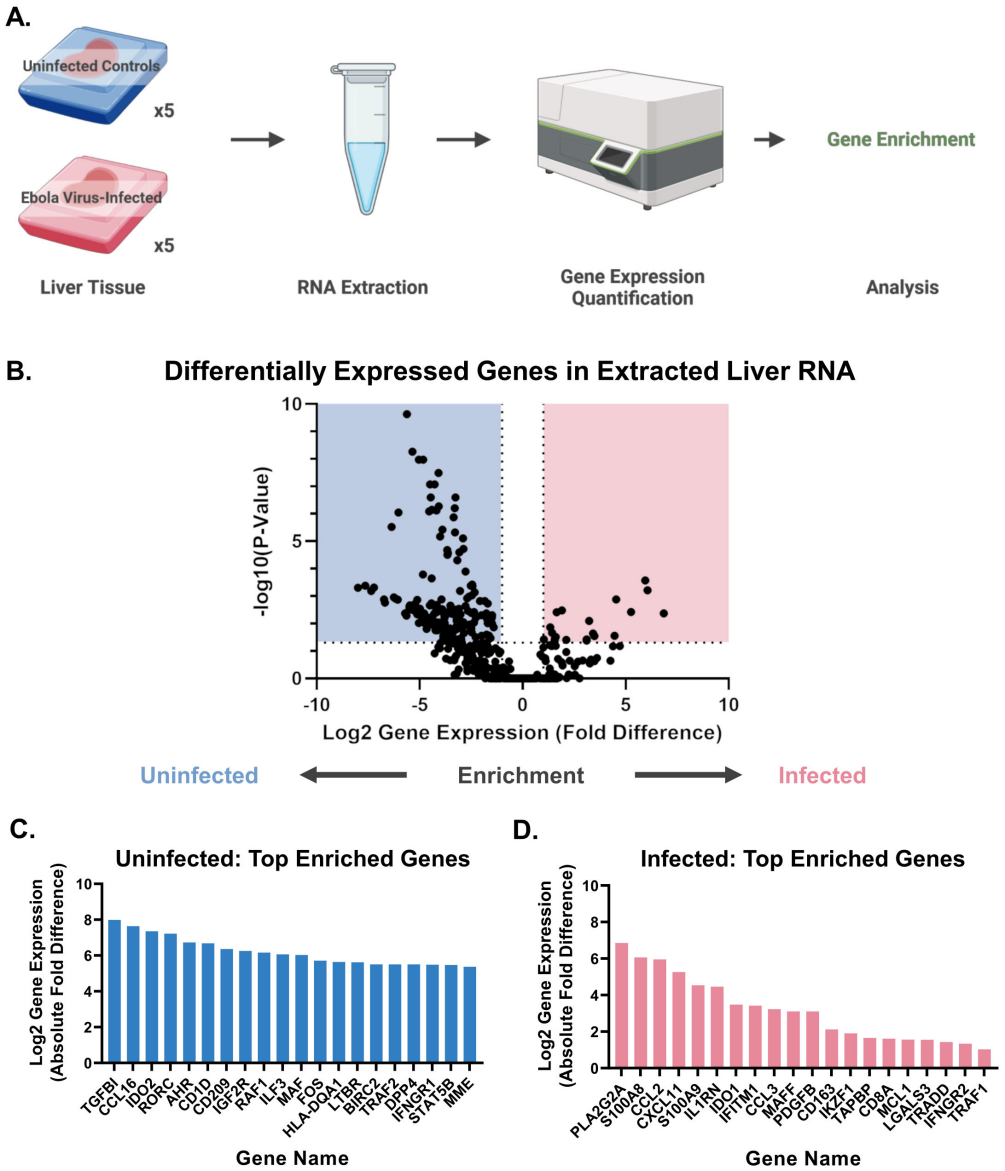
EBOV-infected and uninfected macaques show differential gene expression. **(A)** Workflow for the extraction of RNA from formalin-fixed, paraffin-embedded liver tissue for nCounter analysis. **(B)** Volcano plot of genes enriched in uninfected or infected macaques. Linear regression was performed with Benjamini-Yekutieli correction for multiple comparisons (p<0.05). **(C)** Top 20 genes enriched in uninfected macaques. **(D)** Top 20 genes enriched in infected macaques. Groups: EBOV-infected macaques (n=5), uninfected macaques (n=5). Created in Biorender.com.

We also quantified the expression of immune-related genes in purified RNA from the livers of the EBOV-infected and uninfected macaques. We used a non-human primate immunology nCounter Sprint panel, which measures gene expression by multiplex hybridization with complementary nucleic acid probes. As these tissues were unperfused, gene expression from both the parenchyma and circulatory spaces was captured. We observed genes that were enriched in either the infected or uninfected groups (**Figure 4**). Among the top 20 genes enriched in each group, several of these genes were related to macrophages. In the uninfected group, this included: *IDO2* (39), *CD209* (40), and *HLA-DQA1* (41). In the EBOV-infected group, this included: *S100A8* (Mac387) (42–45), *CCL2* (46), *S100A9* (Mac387) (42–45), *IDO1* (47), *CD163* (48), and *LGALS3* (Galectin-3) (49).

### Region-specific gene and protein expression in liver and lung

**Figure 5:**
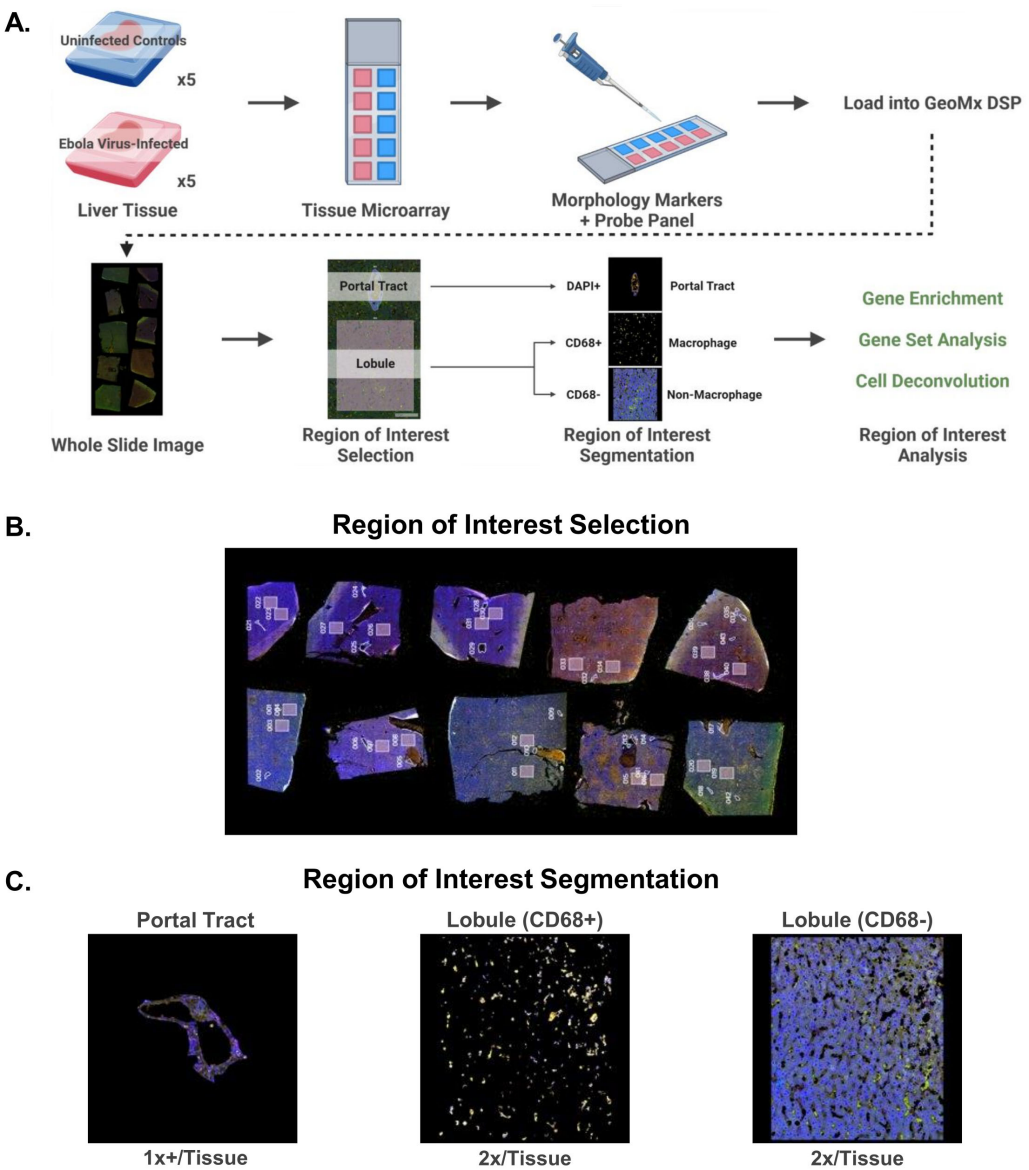
In situ protein and whole transcriptome panel analysis of liver tissue, including macrophages, from uninfected and EBOV-infected cynomolgus macaques using GeoMx Digital Spatial profiling technology. **(A)** Outline of sample preparation, GeoMx DSP procedure, and data analysis. **(B)** Whole slide scan of tissue microarray showing the regions of interest selected for analysis on each tissue. **(C)** Representative images of region of interest segmentation for GeoMx DSP analysis. Created in Biorender.com.

We used GeoMx DSP to quantify gene and protein expression in specific regions of the livers of uninfected and control macaques. This method allows for the detection of gene or protein expression directly from a tissue section using antibody- or nucleic acid-based probes. We took formalin-fixed, paraffin-embedded (FFPE) tissue from the EBOV-infected and control groups and embedded these tissues into a single paraffin block to create a tissue microarray. We then stained sections from this microarray with either an immuno-oncology protein panel or a whole transcriptome panel. These slides were then loaded onto the instrument for region of interest selection based on manual tissue region outlining and marker expression thresholding on our selected fluorescent morphology markers (CD68, PanCK, SYTO83) (**Figure 5A**). At least one portal tract and two lobular areas per macaque were selected as regions of interest for expression analysis. It is important to note that the GeoMx DSP captures average expression data for entire regions of interest, not single-cell level expression. In order to capture macrophage-specific data from the liver lobule, where most hepatic macrophages reside, the lobular regions of interest were segmented into the macrophage (CD68^+^) and non-macrophage (CD68^-^) areas for separate analysis (**Figures 5A–C**). Lung tissue was also analyzed using GeoMx DSP, with the selection of alveolar, blood vessel/neighboring airway, and lymphocyte accumulation regions of interest (**Supplementary Figure S5**).

### Cell deconvolution and M1/M2 polarization in liver and lung

**Figure 6:**
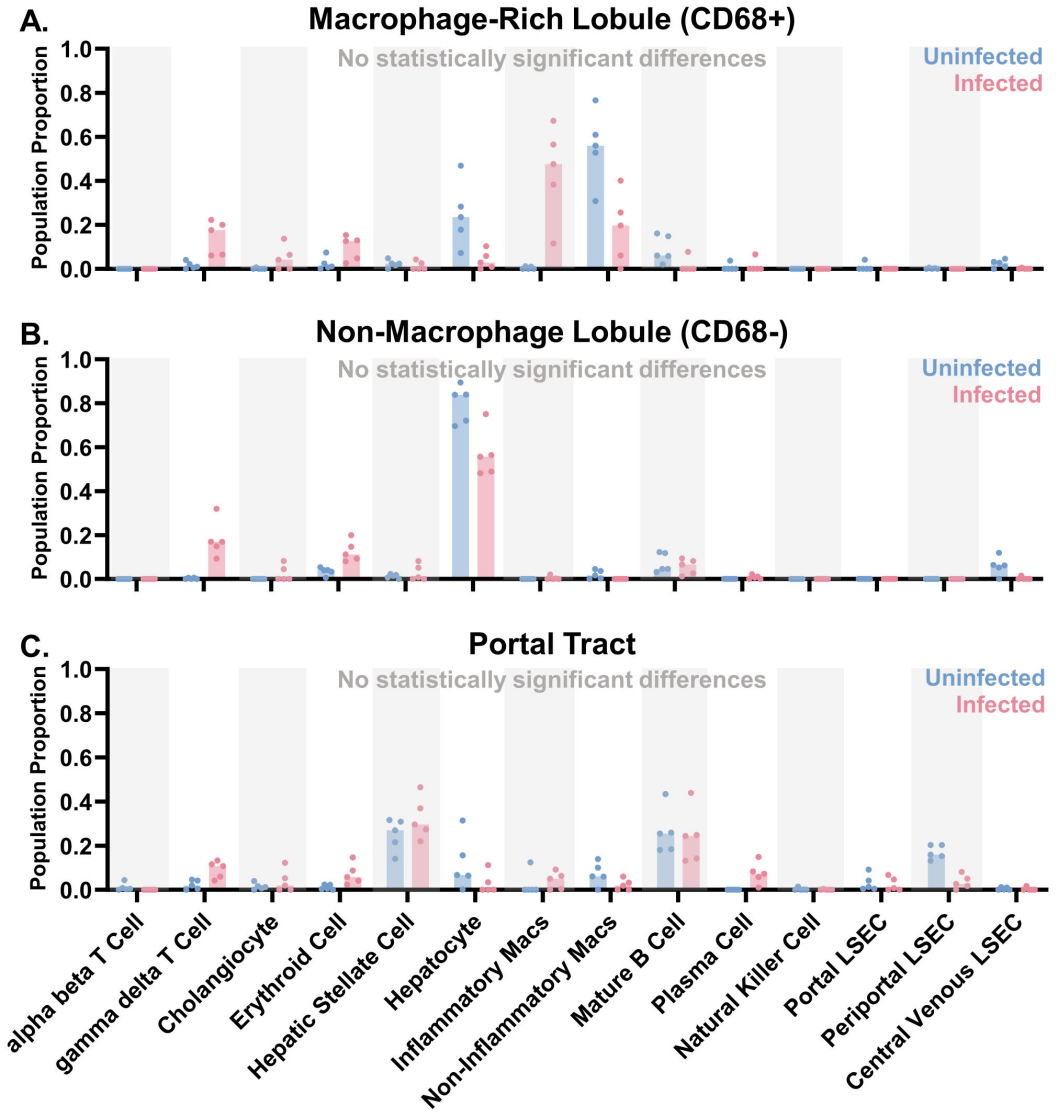
Liver region of interest populations predicted by cell deconvolution in uninfected and infected macaques. Cell deconvolution of the whole transcriptome GeoMx data using a normal human liver cell library was performed for the **(A)** macrophage (CD68^+^) portion of the lobular regions of interest, **(B)** non-macrophage (CD68^-^) portion of the lobular regions of interest, and **(C)** portal tract regions of interest. Median plotted with data points. Mann-Whitney Test with Benjamini, Krieger, and Yekutieli correction for multiple comparisons (False Discovery Rate: 5%) was performed (no statistically significant differences identified, threshold of p<0.05). Groups: EBOV-infected (n=5) and uninfected macaques (n=5).

To confirm that our region-specific analyses were indeed focused on the proper cell types, we performed a cell deconvolution analysis to estimate the cellular populations in the lobular (CD68^+^ and CD68^-^) and portal tract regions of interest. Mixed inflammatory and non-inflammatory macrophages were predicted in the infected group and the predomination of non-inflammatory macrophages in the uninfected group (**Figure 6A**). In the non-macrophage areas of the liver lobule (CD68^-^), hepatocytes dominated, with a trend towards fewer hepatocytes in the infected group, which is consistent with the fact that hepatocytes quantitatively dominate the liver parenchyma (**Figure 6B**). Lastly, the portal tract regions of interest contained several cell types, including hepatic stellate cells and mature B cells in both uninfected and EBOV-infected macaques (**Figure 6C**). No statistically significant differences in the predicted populations between the uninfected and infected groups were identified in any of the regions of interest (individual variability may contribute to this). The fact that macrophages were the most common cell population in the CD68^+^ areas of the lobular regions of interest confirms effective segmentation of these cells. This was further confirmed by the absence of macrophages in the CD68^-^ areas of the liver lobule.

We further evaluated macrophage-related gene expression using a set of genes related to M1 and M2 macrophages (14, 50–52). RNA expression from homogenized liver showed that EBOV infection alters the expression of multiple M1- and M2-related genes, including increases in the expression of IL1-beta, IL-6, and TNF (M1 genes) as well as CLEC4A, CD163, and IL-10 (M2 genes). Alterations in antiviral genes (IRF1, IRF4, IRF7, OAS2, OASL) were also observed. In contrast, RNA expression from CD68^+^ hepatic macrophages in the liver lobule showed no statistically significant differences in M1/M2 gene expression between uninfected and infected macaques (**Supplementary Figure S6**).

We also performed a cell deconvolution analysis of the lung whole transcriptome data to estimate the cellular populations in the alveolar, blood vessel/airway, and lymphocyte accumulation regions of interest. The two cell types with the highest population proportions, alveolar epithelial cell type 1 and blood vessel cells, are consistent with the capillary/small airway architecture of the alveoli (**Supplementary Figure S7A**). In the blood vessel/airway regions of interest, type 1 alveolar epithelial cells, blood vessel cells (trend toward reduced population in infected group), and muscle cells were present, consistent with what would be expected based on the tissue architecture, which consists of both airway tissue as well as larger blood vessels. B cells were also among the most predominant cell types in these regions of interest (**Supplementary Figure S7B**). Lastly, the lymphocyte accumulation regions of interest were predominated by B cells (**Supplementary Figure S7C**). No statistically significant differences in the predicted populations between the uninfected and infected groups were identified in any of the regions of interest (individual variability may contribute to this). Additionally, no statistically significant differences in M1- nor M2-related genes were identified in the alveolar regions of interest, where macrophages reside (**Supplementary Figure S8**).

### Gene set enrichment analysis in liver and lung

**Figure 7:**
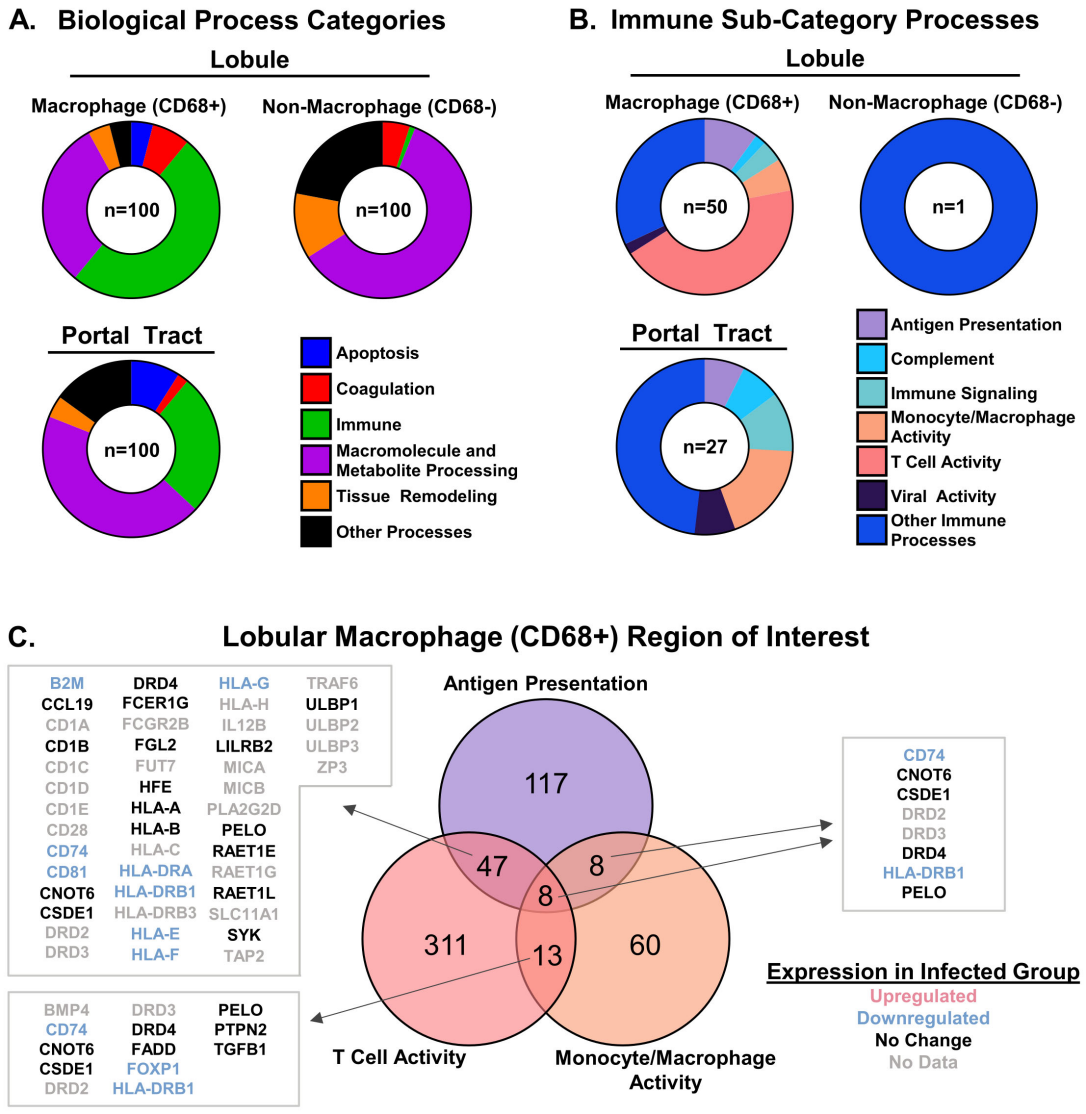
Differential expression of antigen presentation, monocyte/macrophage, and T cell gene sets in hepatic macrophage (CD68^+^) regions of interest in EBOV-infected macaques. **(A)** Differentially expressed gene set categories by region of interest type identified by Global Test analysis (the numbers in the center of each circle represent the total number of differentially expressed gene sets). **(B)** Differentially expressed gene set sub-categories within the Immune category. Results of Global Test analysis within the Immune category (the numbers in the center of each circle represent the total number of differentially expressed gene sets) **(C)** Differentially expressed genes within the antigen presentation, monocyte/macrophage differentiation, and T cell activity gene set categories in the lobular macrophage (CD68^+^) region of interest (No Data: not measured in GeoMx assay but present in Gene Ontology gene sets). Individual gene color coding was done according to the results of the GeoMx whole transcriptome analysis comparing EBOV-infected macaques (n=5) to uninfected macaques (n=5) (see **Figure 8**).

A gene set enrichment analysis was performed to determine what kinds of pathways were altered in the hepatic and pulmonary regions of interest using the Global Test method. Each gene set in the analysis included multiple genes related to a specific biologic process. The Global Test method uses statistical analysis to identify gene sets with a general pattern of gene upregulation or downregulation. The top 100 differentially expressed gene sets between the uninfected and infected macaque groups covered a number of categories, including apoptosis, coagulation, the immune response, metabolic processes, tissue remodeling, and other processes (**Figure 7A**). In the case of the CD68^+^ hepatic macrophages, gene sets for immune-related processes dominated the expression profiles. Sub-categorization of these processes revealed that pathways involved in antigen presentation, complement, immune signaling, monocyte/macrophage differentiation, T cell activity, and viral activity were differentially expressed (**Figure 7B**). Sub-analysis of the antigen presentation, monocyte/macrophage differentiation, and T cell activity highlighted shared down-regulated genes, including class II HLA alleles (**Figure 7C**).

The top 100 differentially expressed gene sets between lung tissue in the uninfected and infected macaque groups also covered the same set of categories (**Supplementary Figure S9**). A number of immune-related gene sets were differentially expressed. In addition to alterations in antigen presentation, monocyte/macrophage activity, and T cell activity, shifts in immune signaling gene sets were common. Few genes shared among the antigen presentation and monocyte/macrophage activity gene sets in the alveoli were differentially expressed.

### Gene and protein enrichment analysis in liver and lung

**Figure 8:**
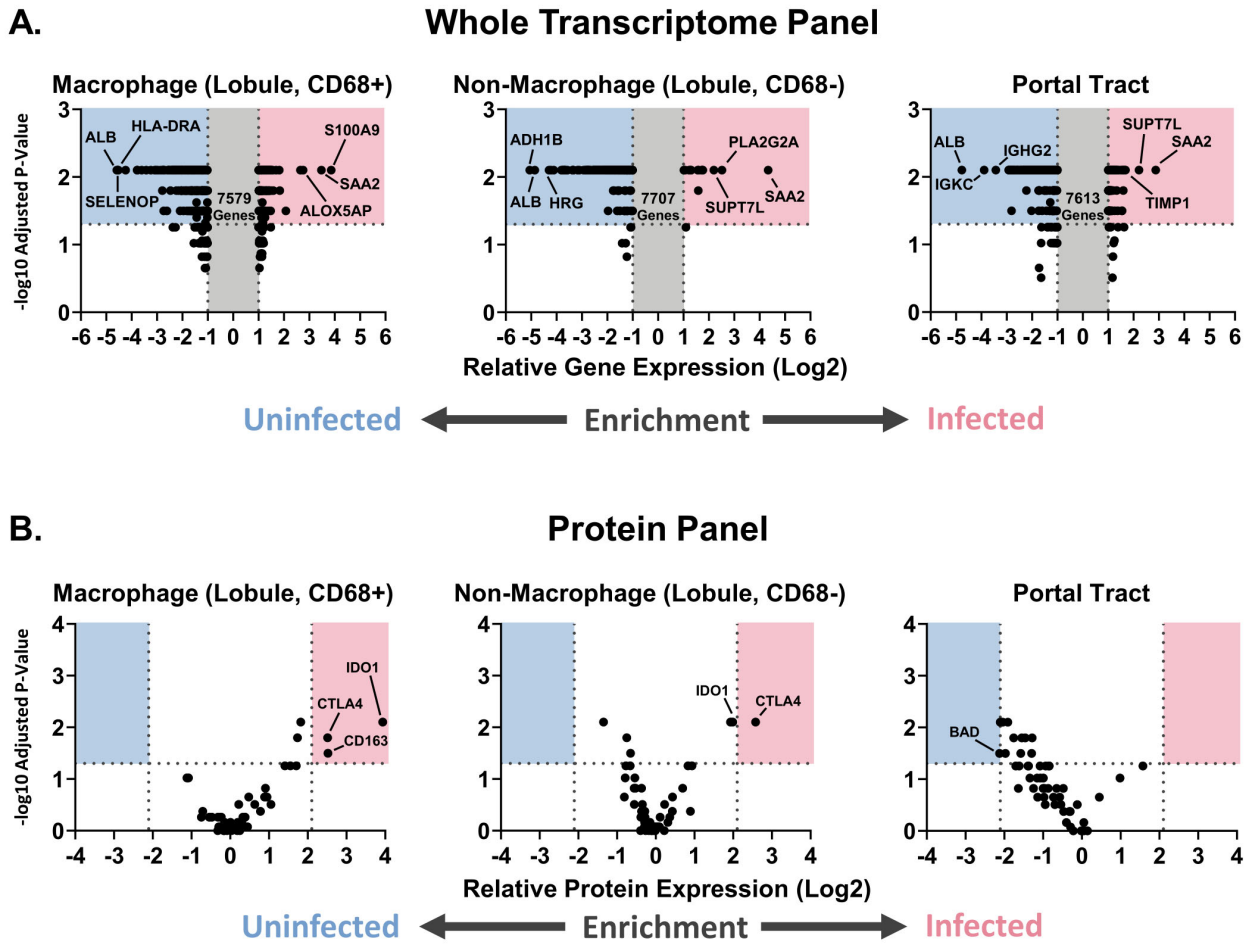
Identification of macrophage and T cell-related genes/proteins enriched in the liver of EBOV-infected cynomolgus macaques for which there are commercially available therapeutics. **(A)** Volcano plot of genes enriched in uninfected or infected macaques, with the three most enriched genes being labelled in each category, from the whole transcriptome analysis. **(B)** Volcano plot of proteins enriched in uninfected or infected macaques. Mann-Whitney test with Benjamini, Krieger, and Yekutieli correction for multiple comparisons (False Discovery Rate: 5%) (p<0.05) performed on the regions of interest from macaques in the uninfected and infected groups (for the whole transcriptome panel, only genes exceeding the relative expression thresholds were statistically analyzed). Groups: EBOV-infected macaques (n=5), uninfected macaques (n=5).

Lastly, we performed single gene enrichment analysis of both the whole transcriptome and protein panel to identify additional genes or proteins altered in infected vs uninfected macaques. Across all three hepatic regions of interest, CD68^+^ lobule, CD68^-^ lobule, and portal tract, some of the top genes enriched in the infected group included *S100A9, SAA2, ALOX5AP*, and *PLA2G2A*. Top genes enriched in the uninfected group included *SELENOP, ALB, HLA-DRA, ADH1B, HRG, IGKC*, and *IGHG2* (**Figure 8A**). In the case of the protein panel, three proteins were identified as enriched in CD68^+^ macrophages in the liver lobule in infected macaques: CD163, IDO1, and CTLA-4. CTLA-4 was also enriched in the non-macrophage portion of the lobule (**Figure 8B**). While IDO1 was significantly enriched in the non-macrophage portion of the lobule, it was just outside the enrichment threshold set to remove housekeeping proteins. BAD was enriched in uninfected macaques in the portal tract regions of interest. While we observed enrichment of *S100A9* at the gene level, the antibody against the protein expressed by this gene was not included in the GeoMx protein panel used in this experiment. However, the expression of this protein (Mac387) was upregulated in the liver of EBOV-infected macaques, as measured by spectral imaging microscopy (**Figure 3**).

Among the lung regions of interest, CD163, a macrophage-related protein, was enriched in the alveolar and blood vessel/airway regions of interest at the protein level in infected macaques (**Supplementary Figure S10**). IDO1 was also enriched in infected macaques in the alveolar and blood vessel/airway regions of interest. Enrichments in uninfected macaques included BCLXL, in the alveoli and blood vessel/airway regions of interest, and BCL6, in the blood vessel/airway regions of interest alone.

In summary, the data recorded in this study highlight a mixed inflammatory/non-inflammatory profile in the hepatic macrophage population, with a paucity of M1- and M2-related gene expression, despite mixed M1/M2 expression in the liver as a whole, in EBOV-infected macaques. These macrophages showed differential expression of monocyte/macrophage differentiation, antigen presentation, and T cell activation gene sets, which were associated with decreased MHC-II allele expression. Clinically relevant proteins, including CD163, IDO1, and CTLA-4, were also upregulated in this population. While CD163 expression was upregulated in the lung, the alveolar areas likewise demonstrated a lack of M1- and M2-related gene expression in infected macaques compared to uninfected controls.

## Discussion

In this study, we assessed the activation profile of macrophages within the liver and lung to better understand the role of these cells in orthoebolavirus infection. This conjunctivally challenged cynomolgus macaque model replicates historical findings in primate models of Ebola virus disease, including histopathological findings, macrophage accumulation, and macrophage association with virus antigen in these tissues (11, 53).

Hepatic macrophages in terminal EBOV-infected cynomolgus macaques display a unique, mixed inflammatory profile compared to previous *in vitro* and primate studies. Human monocyte-derived macrophages are known to produce multiple inflammatory cytokines, including TNF-α, when infected by EBOV (12). In this study, while the liver microenvironment in the infected macaques was broadly characterized by a mixed profile of M1/M2 gene expression relative to controls, the CD68^+^ hepatic macrophage population in the EBOV-infected macaques did not show any significant upregulation nor downregulation of M1- or M2-related genes, including *TNF* and *IL1B*. This was observed despite the prediction of a mixed inflammatory/non-inflammatory macrophage population by cell deconvolution. Furthermore, reduced antigen presentation-related gene expression was identified in this same population by gene set enrichment analysis, including lesser expression of MHC-II alleles. This finding is intriguing as hepatocytes in EBOV-infected rhesus macaques express increased levels of MHC-II protein at the terminal stage of disease (18). These findings suggest that, while macrophages respond in an inflammatory manner to EBOV in *in vitro* studies, *in situ* analysis shows that hepatic CD68^+^ macrophages are not the drivers of M1- nor M2-related gene expression within the liver at the terminal stages of EBOV-infection in cynomolgus macaques. One potential contributing factor to the apparent discrepancy between the presence of inflammatory macrophages within the liver lobule predicted by cell deconvolution and the lack of M1/M2-related gene expression differences in infected vs uninfected macaques may be the cell deconvolution phenotype definition used in this study: inflammatory macrophages (*S100A8, S100A9, LYZ, HLA-DPB1*) and non-inflammatory macrophages (*CD5L, MARCO, VSIG4*). While this inflammatory macrophage population is reported to be associated with greater TNF-α secretion than the non-inflammatory macrophage population, it is possible that these two populations do not overlap one-to-one with macrophage populations classified by traditional markers, such as CD14 and CD16 (54). In a rhesus macaque model of Ebola virus disease, a shift from CD16^+^ to CD14^+^ macrophages was observed in the liver (18). Another potential contributing factor is macrophage degeneration, which has been previously observed histologically in EBOV-infected cynomolgus macaques and humans (55, 56). It is also possible that EBOV, which is known to suppress other antigen-presenting cells, such as dendritic cells, may also suppress macrophage activity late in the infection (57). Future *in situ* work profiling hepatic macrophages at the early and late stages of the disease is warranted to determine how macrophage-specific expression of M1- and M2-related genes varies over the course of the disease. Such work would help harmonize the historic *in vitro* data, which reflect acute cellular responses to infection, with the terminal *in situ* cellular response described in this study.

The expression of CD163 by CD68^+^ hepatic macrophages in EBOV-infected macaques builds upon previous work highlighting this clinically relevant marker. EBOV-antigen-positive CD163^+^ hepatic macrophages have been observed in human cases. In this study, and another published study, CD68^+^ hepatic macrophages co-localized with EBOV antigen (24). Additionally, the soluble form of CD163, associated with macrophage activation syndrome, is known to be elevated in human cases of Ebola virus disease and Sudan virus disease, including severe or fatal infections (25). In EBOV-infected rhesus macaques, hepatic CD163 expression decreased over time compared to control tissue, with a reported transition from CD14^-^CD16^+^CD68^+^CD163^+^ macrophages to CD14^+^CD16^-^CD68^+^CD163^-^ (18). In this study, we observed that hepatic CD68^+^ macrophages have upregulated CD163 protein expression in terminal EBOV-infected macaques compared to uninfected macaques. Future work quantifying CD163 expression in the liver in addition to CD68 over the course of the infection in the cynomolgus macaque model used in this study would help clarify if the same pattern is present in both models or if these represent distinct immunologic responses. Correlating the timeline of CD163 expression by hepatic macrophages with circulating soluble CD163 would also shed light on whether these populations are the source of this circulating biomarker.

In addition to this biomarker, the hepatic microenvironment generally, and CD68^+^ hepatic macrophages in particular, express multiple genes and proteins associated with macrophage-targeting molecules already under study for the treatment of other liver diseases, as well as cancer. Previous studies in orthoebolavirus-infected macaques have shown increases in hepatic macrophage populations (58, 59). S100A8 and S100A9 (also known as MRP8 and MRP14, respectively) are associated with recently recruited systemic macrophages (42, 43, 60). Accumulation of leukocyte antigen L1-expressing cells (another name for the S100A8/S100A9 dimer Mac387) in Reston virus (RESTV)-infected macaques has been previously shown (61, 62). In our study, the enriched expression of *S100A8* and *S100A9* by CD68^+^ hepatic macrophages in EBOV-infected macaques indicates a similar increase in recently recruited macrophages at the terminal stage of disease compared to uninfected controls. Dual CCR2/CCR5 inhibition, studied in the treatment of Metabolic Dysfunction Associated Steatotic Liver Disease, may help reduce this population by impairing monocyte recruitment into the liver (46). Additionally, previous drug discovery work for Ebola virus disease therapeutics has suggested IDO1, which is expressed by macrophages and modulates T cell activity, as a potential drug target (63). The expression of IDO1, and another immune checkpoint protein, CTLA-4, by CD68^+^ hepatic macrophages suggests that these cells play a role in immune tolerance during terminal disease. Established therapies inhibiting the action of these molecules could help lift this immune tolerance (64, 65). Lastly, Galectin-3 is thought to promote hepatic inflammation in fatty liver disease via TLR-4-mediated NLRP3 inflammasome activation (66). In this study, Galectin-3 expression was upregulated in the homogenized liver of EBOV-infected macaques. As such, therapeutics targeting this molecule may provide an avenue for modulating inflammatory macrophage activities in the liver in Ebola virus disease (67). Taken together, multiple existing therapeutics have the potential to augment the immune response to EBOV infection, expanding the therapeutic strategies beyond virus-specific biologics.

Although lung pathology is less prominent in Ebola virus disease, certain findings paralleled those in the liver, while others were distinct. Overall, fewer significant differences between EBOV-infected macaques and uninfected controls were observed. CD163, upregulated on hepatic CD68^+^ macrophages, was upregulated in both the alveolar and blood vessel/airway regions of the lung. While macrophages were not specifically segmented in the GeoMx analysis of the lung, this upregulation indicates that pulmonary macrophages may be yet another source of soluble CD163, associated with macrophage activation syndrome (25). The upregulation of IDO1 in these same regions in the infected group also indicates that macrophage-mediated immunomodulation may be a systemic process in terminal disease rather than something limited to the liver. Interestingly, CCR2, a chemotactic receptor, is downregulated in EBOV-infected macaques in the lung, but not in the liver (68). This may suggest reduced recruitment of systemic immune cells to the lung, which is consistent with the relative lack of histopathological findings in lung compared with the liver.

This study has several limitations that were considered in the design and analysis of the presented experiments. The tissues for the uninfected and infected macaque groups were sourced from different labs and included five animals per group. We minimized variation between the source animals by using sex-matched control tissues to ensure the same number of male and female subjects were included in each group. Another limitation of the control group was the inclusion of non-naive macaques (clinical histories including *Balantidium*, SRV, B virus, STLV-1, and/or measles) (**Supplementary Table S1**). Differences in the formalin fixation time between the uninfected and infected macaque groups may have affected the quantity and quality of RNA in these tissues (69). In the nCounter experiment, this difference was mitigated by correcting the amount of sample loaded based on RNA quality and quantity assessments using Qubit, Nanodrop, and Bioanalyzer. Additionally, in both the RNA and protein expression experiments with the GeoMx DSP as well as the nCounter experiment, the data were normalized to correct for variations in sample loading. A limitation of the GeoMx platform, compared to other high-plex spatial analysis platforms, is that this method captures average expression across a region of interest, not single-cell expression. In order to capture macrophage-specific data, we divided the liver lobule regions of interest into two separate collections, one for the areas expressing CD68 and one for the areas negative for this same marker. We did not collect macrophage-specific data in the lung using the GeoMx DSP due to the limit on the number of samples the instrument can collect in one run. We thus prioritized our region of interest selection to the liver over the lung given that the liver has a greater pathological burden in Ebola virus disease. An additional limitation of the GeoMx and nCounter panels is that they do not contain probes for viral RNA or protein. Instead, we included an anti-VP35 antibody as part of the spectral imaging microscopy panel to assess the presence of viral antigen within the tissues. A final limitation of this study is the lack of spatial analysis between the location of virus antigen and macrophages in these tissues. Recent updates to the Visiopharm software include more effective tools for assessing spatial relationships between stains in a tissue. As such, analyses of this kind would be valuable to include in future studies.

In summary, we demonstrated that the CD68^+^ hepatic macrophages in terminal EBOV-infected macaques include a mix of inflammatory and non-inflammatory phenotypes that express CD163, a biomarker associated with macrophage activation syndrome, but lack significant expression of many traditional M1 and M2-related genes. This, in conjunction with the expression of IDO1, CTLA-4, and downregulated expression of MHC-II alleles, suggests that CD68^+^ hepatic macrophages may not play a monolithic inflammatory role at the terminal stage of the disease. As IDO1 expression is also enriched in the lung, these findings may not be limited to just the liver. Furthermore, the upregulated expression of multiple genes and proteins targeted by therapeutics from the fields of inflammatory liver disease and cancer offer opportunities for new immunomodulatory strategies for the treatment of Ebola virus disease. While the results of this study are limited to gene and protein expression correlations with the known activities of these molecules, this is the first study to use the high-plex, region-specific GeoMx DSP instrument to analyze liver and lung tissue expression in EBOV-infected macaques, with a particular focus on macrophages. Future work studying the time course of macrophage activity, from early to late stages, and the disease outcomes of modulating targetable genes and proteins will clarify their contribution to disease severity and survival.

## Materials and methods

### Non-human primate samples

The data presented in this study were generated from the analysis of historical samples collected from a previously published primate study (38). We include here a summary of key parameters of the study from which these historical samples were collected. Three male and two female cynomolgus macaques were challenged on Day 0 with 10^4^ plaque-forming units of EBOV Kikwit via the conjunctival route. Body weight, temperature, whole blood, and serum samples were collected at Day -7, Day 0, Day 3, Day 6, Day 9, and at the time of euthanasia. Viral load, albumin, alanine aminotransferase, and total bilirubin tests were performed on the serum samples. Liver and lung tissues were collected at the time of euthanasia. The collected tissues were placed in formalin for long-term storage.

For this study, the formalin-fixed tissues were recovered from long-term storage and were processed using a Tissue-Tek VIP 6 AI Vacuum Infiltration Processor (Sakura Finetek USA, Inc., Torrance, CA) and paraffin-embedded.

Formalin-fixed (48-72 hours), paraffin-embedded liver and lung tissues from sex-matched control cynomolgus macaques (3 males and 2 females) were received from the Southwestern National Primate Research Center, San Antonio, TX, USA (See **Supplementary Table S1** for infection-related clinical history).

### Spectral imaging microscopy

Cynomolgus macaque liver and lung tissue sections were stained using the Ventana Discovery Ultra automated stainer (Roche Diagnostics, Indianapolis, IN). The panel consisted of a four-marker panel (**Supplementary Table S2**).

Images of the stained slides were captured using a Vectra 3 microscope (Akoya Biosciences, Marlborough, MA) and InForm software (Akoya Biosciences, Marlborough, MA). The images were imported into Visiopharm (Hoersholm, Denmark) and analyzed using custom algorithms.

### GeoMx digital spatial profiling

#### Tissue embedding

Formalin-fixed liver and lung tissues from the NHP study were processed using a Tissue-Tek VIP 6 AI Vacuum Infiltration Processor (Sakura Finetek USA, Inc., Torrance, CA) and paraffin-embedded. Square pieces of these embedded tissues and the control tissues were cut from the blocks. The paraffin was melted from these pieces. The pieces were then paraffin-embedded into a single block.

5µm serial sections of the liver and lung blocks for the NHP study were cut and shipped overnight to the NanoString Technology Access Program (Seattle, WA) for analysis.

#### Run instrument

The NanoString Technology Access Program ran the submitted liver samples according to the following parameters:

##### Whole transcriptome assay

The liver and lung slides were stained with the Human Whole Transcriptome Panel (NanoString, Seattle, WA) and fluorescent morphology markers. The slides were then scanned on the GeoMx Digital Spatial Profiler instrument (**Supplementary Table S3**).

Regions of interest were then drawn on the whole slide scans by manual tissue region outlining. For the liver slide, 1 or more portal tracts and 2 lobular regions were selected from each of the tissues on the slide. For the lung slide, 1 alveolar region per tissue, 3-5 blood vessel/airway regions per group, and 2 lymphocyte accumulation regions per group were selected. Segmenting of the regions of interest was also performed (**Supplementary Table S4**).

The Whole Transcriptome Panel probes were collected and counted using Next Generation Sequencing. The probe counts were uploaded into the GeoMx DSP Analysis Suite software (NanoString, Seattle, WA).

##### Immuno-oncology protein panel

The liver and lung slides were stained with the Immuno-Oncology Protein Panel [(v1.0) Human Immune Cell Profiling Protein Core (NanoString, Seattle, WA), (v1.0) Human IO Drug Target Protein Module (NanoString, Seattle, WA), (v1.0) Human Immune Activation Status Protein Module (NanoString, Seattle, WA), (v1.0) Human Immune Cell Typing Protein Module (NanoString, Seattle, WA), (v0.9) Human PI3K/AKT Signaling Protein Module (NanoString, Seattle, WA), (v1.0) Human Pan-Tumor Protein Module (NanoString, Seattle, WA), (v1.1) Human nC Cell Death Protein Module (NanoString, Seattle, WA)] and fluorescent morphology markers. The slides were then scanned on the GeoMx Digital Spatial Profiler instrument (**Supplementary Table S3**).

Regions of interest were then drawn on the whole slide scans by manual tissue region outlining. For the liver slide, 1 or more portal tracts and 2 lobular regions were selected from each of the tissues on the slide. For the lung slide, 1 alveolar region per tissue, 3-5 blood vessel/airway regions per group, and 2 lymphocyte accumulation regions per group were selected. Segmenting of the regions of interest was also performed (**Supplementary Table S4**).

The Immuno-Oncology Panel probes were collected and counted using the nCounter platform (NanoString, Seattle, WA).

#### Data analysis

##### Image export

The liver slide image from the Immuno-Oncology protein panel assay was adjusted to the following render settings to remove background stain (FITC: 1300-50000, Cy3: 800-14856, Texas Red: 500-2000, Cy5: 500-10000). The lung slide image from the Immuno-Oncology protein panel assay was adjusted to the following render settings to remove background stain (FITC: 1000-15000, Cy3: 2000-25000, Texas Red: 600-2800, Cy5: 3000-40000). With all of the channels turned on, the region of interest images were exported as an ROI report with the following settings (Segments: Unchecked).

##### Visiopharm analysis

The images from the GeoMx DSP Immuno-Oncology Protein Panel Assay were imported into Visiopharm. Custom algorithms were run in the following order: ROI Detection, Nuclear Detection, Cell Phenotyping. CD68^+^ cell prevalence data were generated from the cell phenotyping data. Between-groups comparison of the cell prevalence data was performed using a Mann-Whitney test with two-stage step-up method of Benjamini, Krieger, and Yekutieli correction for multiple comparisons (False Discovery Rate: 0.05) using GraphPad Prism (Boston, MA).

##### Gene enrichment

The probe counts were uploaded into the GeoMx DSP Analysis Suite software (NanoString, Seattle, WA). Segment QC, probe QC, target filtering, and normalization were performed on the raw data (**Supplementary Table S5**). While the segment QC, probe QC, and target filtering were performed on the combined liver and lung datasets, the liver and lung data were normalized separately.

Gene enrichment analyses between the control and EBOV-infected groups were performed using a Mann-Whitney test with Benjamini, Krieger, and Yekutieli correction for multiple comparisons (False Discovery Rate: 0.05) for each of the region of interest types, respectively, using GraphPad Prism (Boston, MA).

##### Cell deconvolution

Cell deconvolution of the liver and lung region of interest whole transcriptome data was performed using the SpatialDecon_plugin.R script in the GeoMx DSP Analysis Suite software with the Landscape_Adult_Liver_10x.csv and Lung_plus_neutrophils.csv cell profile libraries, which may be downloaded from the NanoString GeoScript Hub webpage, under the SpatialDecon link (https://nanostring.com/products/geomx-digital-spatial-profiler/geoscript-hub). Between-groups comparisons were performed using a Mann-Whitney test with two-stage step-up method of Benjamini, Krieger, and Yekutieli correction for multiple comparisons (False Discovery Rate: 0.05) using GraphPad Prism (Boston, MA).

##### Global Test

Identification of differentially expressed gene sets was performed on the liver and lung whole transcriptome data for each of the regions of interest, comparing the uninfected versus EBOV-infected groups using the Global Test method in R (see **Supplementary Data** for R script).

##### Protein enrichment

The probe counts were uploaded into the GeoMx DSP Analysis Suite software (NanoString, Seattle, WA). The Data QC, background correction, and target filtering steps were then sequentially performed (**Supplementary Table S6**). While the liver and lung data were QC’ed together, the data from these tissues was separately processed for the background correction and target filtering steps.

Protein enrichment analyses between the control and EBOV-infected groups were performed using a Mann-Whitney test with Benjamini, Krieger, and Yekutieli correction for multiple comparisons (False Discovery Rate: 0.05) for each of the region of interest types, respectively, using GraphPad Prism (Boston, MA).

### nCounter^®^ RNA analysis

#### RNA extraction

RNA was isolated from FFPE liver tissue from the NHP study using the FFPET RNA Isolation kit (Roche, Catalog Number: 06650775001) according to the manufacturer’s instructions. The resulting RNA samples were quantified using Qubit 4 (Invitrogen) and Bioanalyzer (High Sensitivity RNA kit). Most extracted RNA samples had 260/280 ratios between 1.44-1.89 by Nanodrop analysis and a RIN value between 1.0-2.3 by Bioanalyzer analysis.

#### Run instrument

The purified RNA was diluted so that a maximum of 150ng of RNA was loaded into each sample well. The amount of RNA loaded from each sample ranged between 95.8ng-150ng. The RNA loading calculations were based on the average of the concentration measured by Qubit 4 and Bioanalyzer analysis for each sample. In samples where 150ng of RNA could not be achieved, undiluted sample was loaded to maximize the amount of RNA for analysis. These samples were analyzed using the NHP Immunology V2 nCounter^®^ Panel (NanoString, Catalog Number: 115000276) according to the manufacturer’s instructions.

### Data analysis

The probe count data were analyzed using the nSolver 4.0 software (NanoString, Seattle, WA). QC analysis was performed. The probe count data were normalized using the following settings for Positive Control Normalization (Mean Type: Geometric Mean, Threshold Min: 0.3, Threshold Max: 3) and CodeSet Content Normalization for the housekeeping genes (Mean Type: Geometric Mean, Threshold Min: 0.1, Threshold Max: 10).

Gene enrichment analyses between the control and EBOV-infected groups were performed using linear regression with Benjamini-Yekutieli correction (omit low count data: unchecked).

### Histology

Formalin-fixed tissues from EBOV-infected macaques were processed using a Tissue-Tek VIP 6 AI Vacuum Infiltration Processor (Sakura Finetek USA, Inc., Torrance, CA) and paraffin-embedded. The FFPE tissues from both the infected and uninfected macaque groups were sectioned at 5 µm using a HM 325 Rotary Microtome (Thermo Fisher Scientific, Inc., Waltham, MA), and mounted on slides. Hematoxylin and eosin (H&E) staining, dehydration, and coverslip placement were conducted using a Tissue-Tek Prisma Plus Automated Slide Stainer (Sakura Finetek USA, Inc., Torrance, CA). Histological assessment was performed by a board-certified pathologist (HSL).

## Data Availability

The original contributions presented in the study are included in the article/**Supplementary Material**, further inquiries can be directed to the corresponding author/s.
