# Supplementary material for: Hepatic and pulmonary macrophage activity in a mucosal challenge model of Ebola virus disease
Source: Front Immunol. 2024 Nov 20;15:1439971. doi: 10.3389/fimmu.2024.1439971 (PMC11615675; doi:10.3389/fimmu.2024.1439971)
Supplement: Supplementary file 1 [file DataSheet1.zip › Manuscript Suppl.pdf]

Supplementary Material

Hepatic and Pulmonary Macrophage Activity in a Mucosal Challenge Model of Ebola Virus Disease

Timothy G. Wanninger*, Omar A. Saldarriaga, Esteban Arroyave, Daniel E. Millian, Jason E. Comer, Slobodan Paessler, Heather L. Stevenson

*** Correspondence:** Corresponding Author: tiwannin@utmb.edu

1 Table of Contents

Supplementary Tables…………………………………………………………………………...2

Supplementary Figures………………………………………………………………………..6

GlobalTest R Script……………………………………………………………………………16

GeoMx Data

Whole Transcriptome Data – Liver……………...S1 Appendix_WTA Panel_Liver.xlsx

Whole Transcriptome Data – Lung……………....S2 Appendix_WTA Panel_Lung.xlsx

Protein Panel – Liver…………………………..S3 Appendix_Protein Panel_Liver.xlsx

Protein Panel – Lung…………………………...S4 Appendix_Protein Panel_Lung.xlsx

nCounter Data……………………..S5 Appendix_nCounter Relative Gene Expression Data.xlsx

2 Supplementary Tables

Table S1. Infection-related clinical history in uninfected macaques.

Macaque	Clinical History of Infection
6	Measles
7	SRV, Measles
8	Balantidium
9	B virus, Measles
10	STLV, Measles

Table S2. Macrophage and EBOV antigen spectral imaging microscopy panel

Marker	Antibody Clone	Antibody Dilution	Company	Fluorophore	Fluorophore Dilution	Reaction Position
CCR2	7A7	1:200	Abcam	Opal 570	1:150	1
Mac387	Mac387	1:500	Dako	Opal 690	1:100	2
CD68	KP1	Ready to Use	Biogenex	Opal 520	1:100	3
VP35	10C7	1:300	Kerafast	Opal 650	1:100	4

Table S3. Morphology Marker Staining Conditions

Marker	Antibody Clone	Antibody Dilution	Fluorophore	Company	Catalog Number	Detection Channel (Exposure)
CK8/18	KRT8/803+ KRT18/835	1:200	AF488	Novus Biologicals	NBP2-47983AF488	FITC (200ms)
PanCK	AE1/AE3	1:400	AF488	Novus Biologicals	NBP2-33200AF488	FITC (200ms)
CD68	KP1	1:400	AF647	Santa Cruz Biotechnology	sc-20060AF647	Cy5 (300ms)
DNA	SYTO83	1:10	-	Thermo Fisher	S11364	Cy3 (60ms)

Symbols: + Two antibody clones used, ms: milliseconds

Table S4. Region of Interest Segmentation Settings

Tissue	Region of Interest	Segment	FITC Channel	Cy3 Channel	Texas Red Channel	Cy5 Channel	Segment Contents
Liver	Lobule	CD68+	Ø	Ø	Ø	+	Macrophages in lobule
		CD68-	Ø	Ø	Ø	-	Non-macrophage cells in lobule
	Portal Tract	DNA+	Ø	+	Ø	Ø	All cells in portal tracts
Lung	Alveoli	DNA+	Ø	+	Ø	Ø	All cells in alveoli
	Blood Vessel/ Airway	DNA+	Ø	+	Ø	Ø	All cells in blood vessels/airways
	Lymphocyte Accumulation	DNA+	Ø	+	Ø	Ø	All cells in lymphocyte accumulations

Symbols: + Stain included in segmentation area, Ø Channel ignored, - Stain excluded from segmentation area

Table S5. Whole Transcriptome Assay Data Processing Parameters

Step	Parameters	Value
Segment QC	Filter Targets	No
	Use Raw Reads Threshold	Yes
	Raw Reads Threshold	1000
	Use Aligned Reads Threshold	Yes
	Aligned Reads Threshold	80
	Use Stitched Reads Threshold	Yes
	Stitched Reads Threshold	90
	Use Trimmed Reads Threshold	Yes
	Trimmed Reads Threshold	90
	Use Sequencing Saturation Threshold	Yes
	Sequencing Saturation Threshold	50
	Use Negative Probe Count GeoMean Threshold	Yes
	Negative Probe Count GeoMean Threshold	10
	Use No Template Control Threshold	Yes
	No Template Control Count	1000
	Use Surface Area Threshold	No
	Use Nuclei Count Threshold	No
Probe QC	Remove Failed Segments before Executing Probe QC	No
	Use Exclude if Below Probe Ratio Threshold	Yes
	Ratio of Probe GeoMean Across All Segments	0.1
	Use Exclude if Below Grubbs Fail Percent Threshold	Yes
	Percent AOIs Threshold for Grubbs Test	20
	Use Exclude Local Segment Outliers	Yes
	Standard Deviation Amount for the LOQ	2
Target Filtering	Expression Filtering Mode	LOQ
	Filtering by	Target
	Frequency	1
Normalization	Filter Segment	Yes
	Filter Targets	Yes
	Reference Targets	All
	Method	Third Quartile

Table S6. Immuno-Oncology Protein Panel Data Processing Parameters

Step	Parameters	Value
Data QC	Filter Targets	Yes
	Used Binding Density QC	Yes
	Binding Density QC	0.1-2.25
	Used Positive Control Normalization QC	Yes
	Used Positive Control Normalization QC	0.3-3.0
	Used Percent of FOV Detected QC	Yes
	Percent of FOV Detected QC	75.0
	Used Surface Area QC Threshold	Yes
	Surface Area QC Threshold	1600.0
	Used Nuclei Count QC Threshold	Yes
	Nuclei Count QC Threshold	20.0
Background Correction	Filter Segment	Yes
	Filter Targets	Yes
	Background Correction Method	Mean +2
	Average Background Method	Signal to Noise Ratio
	Background Targets	Liver: Rb IgG, Ms IgG1<br>Lung: Ms IgG2a, Rb IgG, Ms IgG1
Target Filtering	Filtered By	Target
	Frequency	1
	User-defined Value	3

3 Supplementary Figures

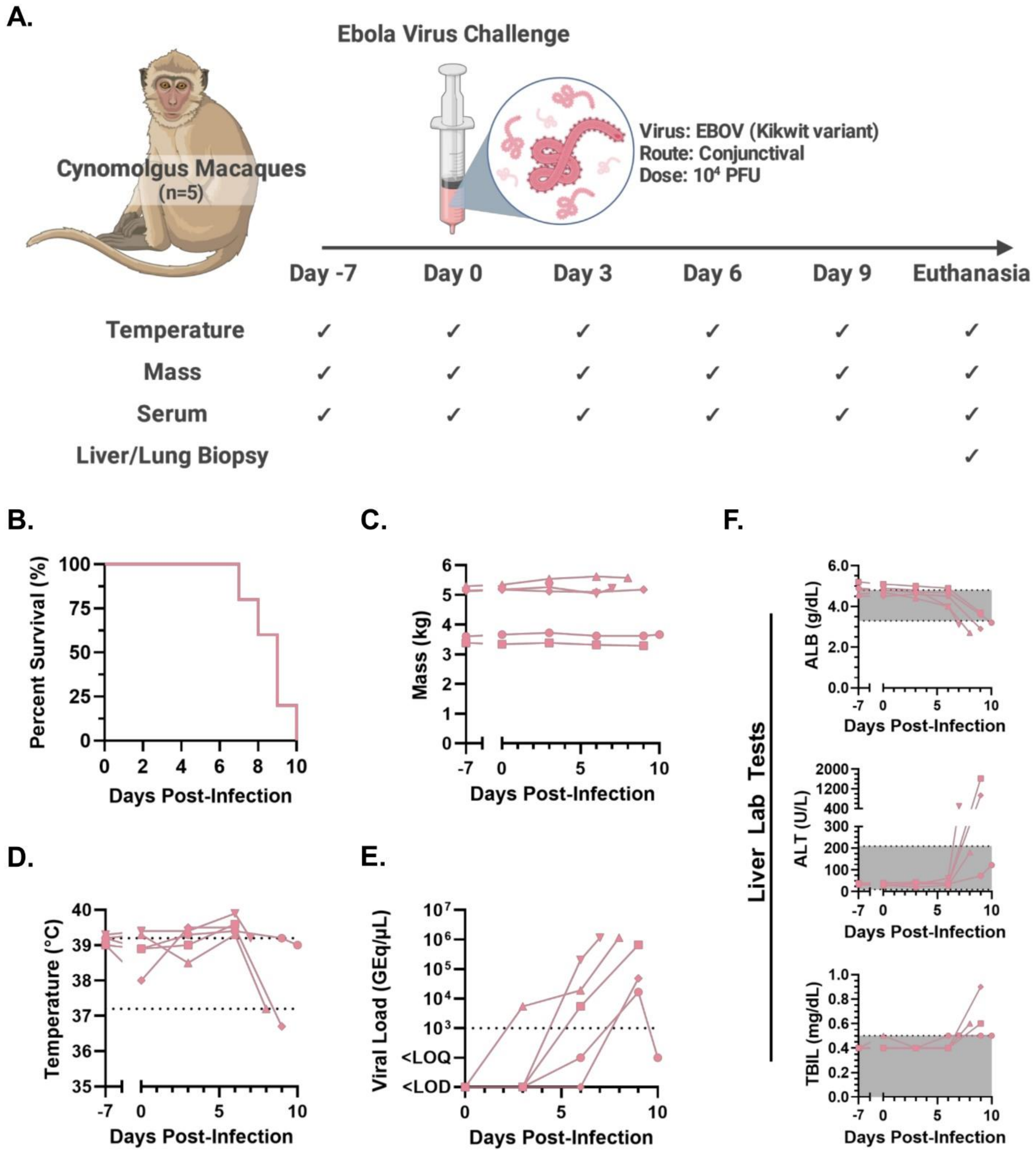

Figure S1. Hypothermia, viremia, and signs of liver injury in EBOV-challenged cynomolgus macaques. A) Flowchart outlining the cynomolgus macaque challenge model challenge conditions and specimen collection. B) Percent Survival, C) Body Weight, D) Body Temperature (dashed lines: normal range), and E) Serum Viral Load (Limit of Quantification: 10^3 Geq/μL, Below Limit of Quantification: <LOQ, Below Limit of Detection (0 Geq/μL): <LOD) by day post-infection for EBOV-challenged cynomolgus macaques. F) Serum markers of liver injury/function by day post-infection in EBOV-challenged cynomolgus macaques. The normal range values (highest and lowest normal range values between males and females) are indicated by the dashed lines and gray shading. Abbreviations: Albumin, ALB; Alanine Aminotransferase, ALT; Total Bilirubin, TBIL. Created in Biorender.com. These historical data are published in [38].

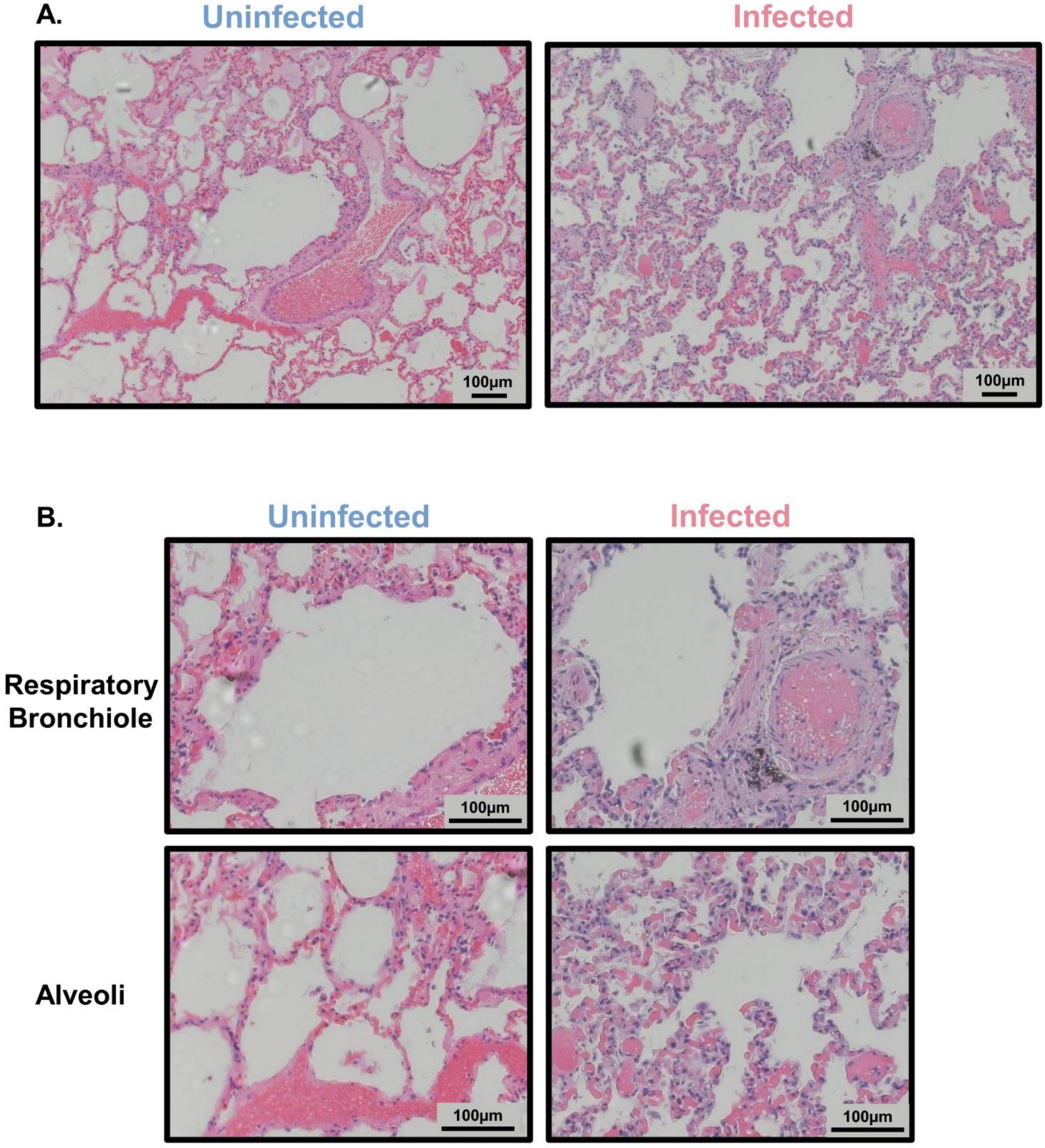

Figure S2. Interstitial mononuclear infiltrate is present in EBOV-infected cynomolgus macaques.
A) Representative low magnification images of hematoxylin and eosin-stained lung from uninfected control and EBOV-infected cynomolgus macaques at the time of euthanasia. B) Representative high-magnification images of hematoxylin and eosin-stained respiratory bronchioles and alveoli from the lungs of uninfected control and EBOV-infected cynomolgus macaques at the time of euthanasia. n=5 macaques/group.

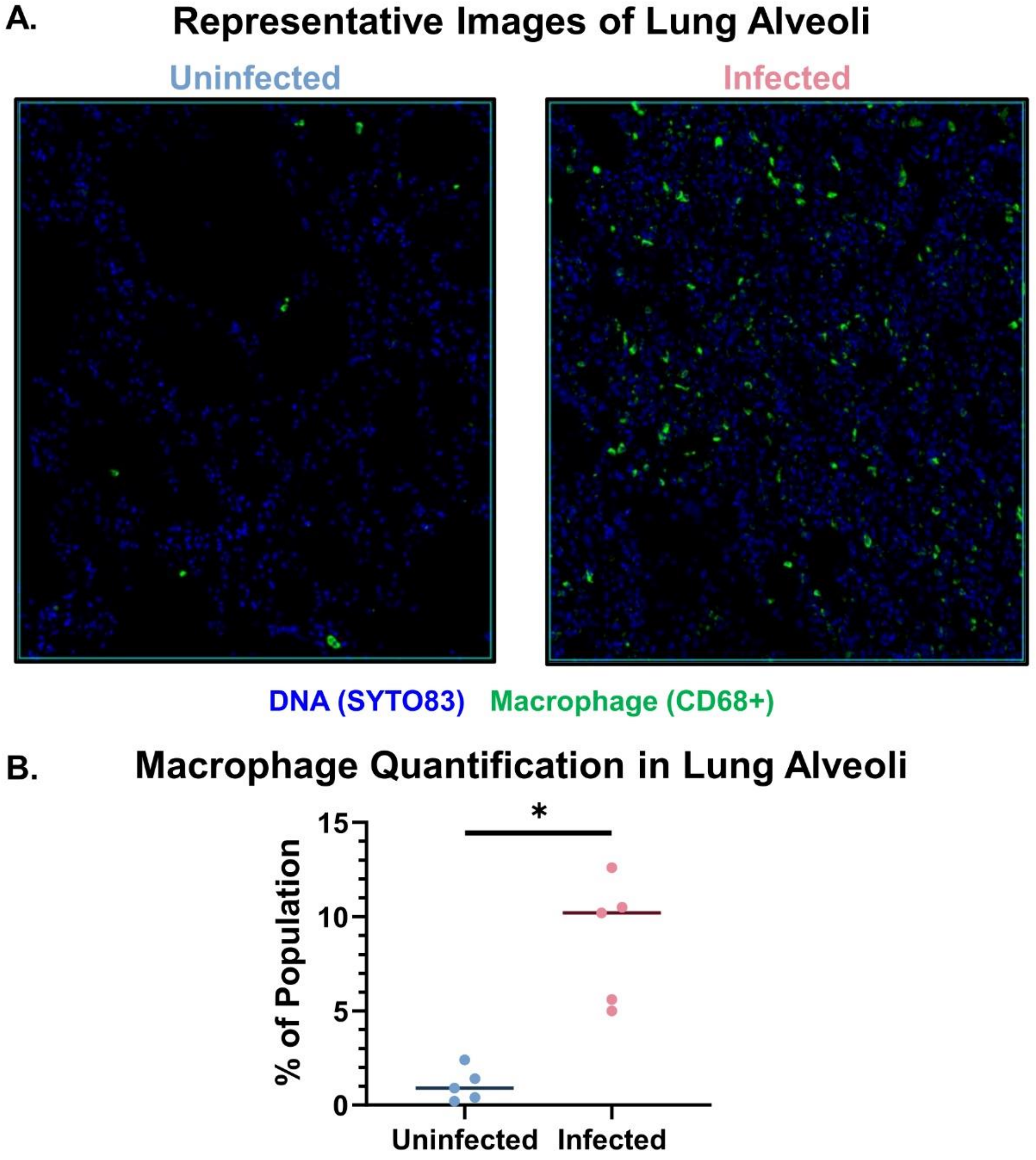

Figure S3. Macrophage accumulation is observed in the lungs of EBOV-infected cynomolgus macaques. A) Representative images of the alveolar regions of interest analyzed using GeoMx DSP in uninfected and infected macaques. These images show macrophages ($CD68^+$ cells, Green) and nuclei (Syto83, Blue). B) Quantification of the percentage of macrophages ($CD68^+$ cells) in the alveolar region of interest populations. Mann-Whitney test with Benjamini, Krieger, and Yekutieli correction for multiple comparisons (False Discovery Rate: 5%) was performed between the groups (EBOV-infected macaques (n=5), uninfected macaques (n=5) (* $p<0.05$).

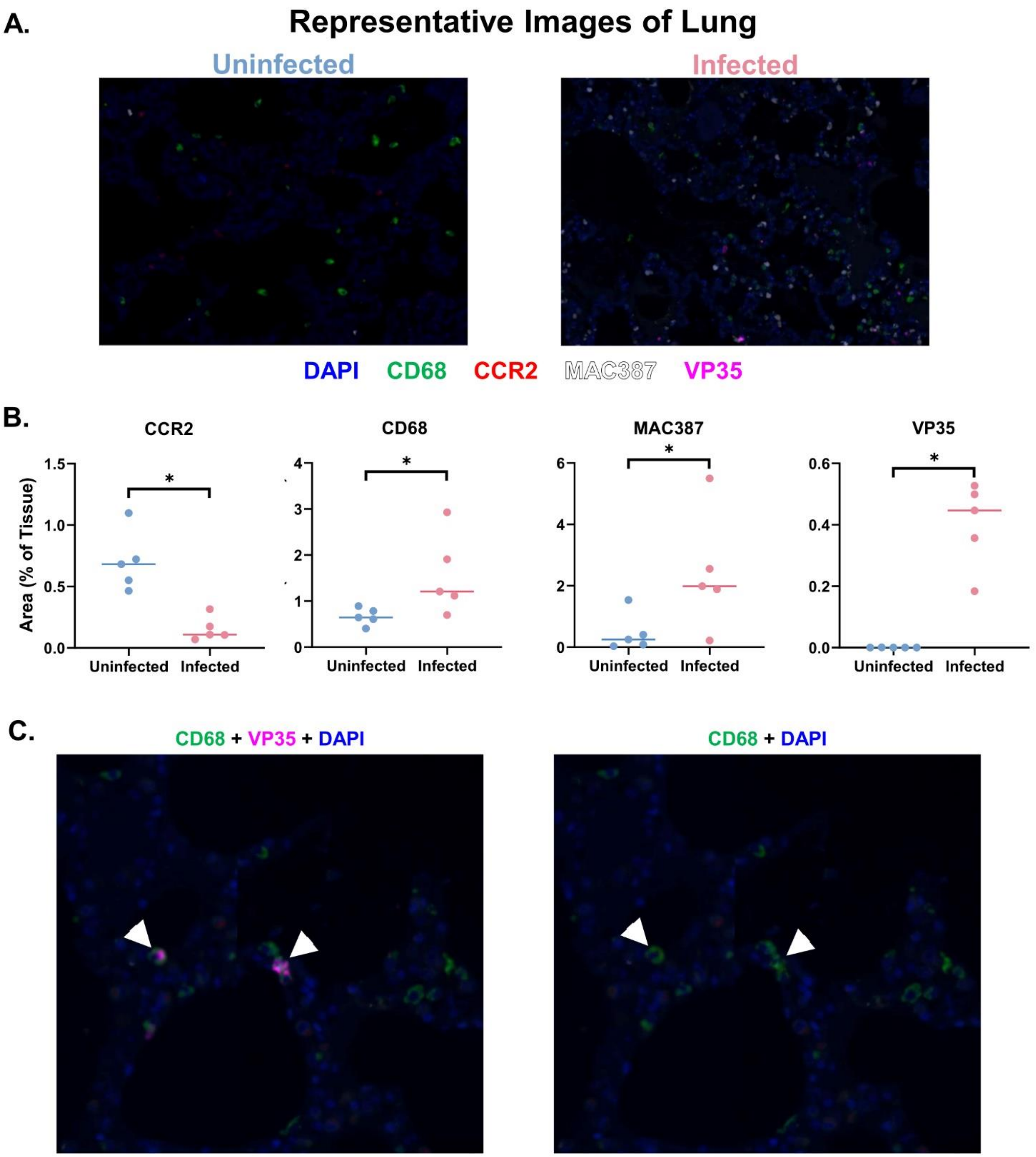

Figure S4. Multiplex spectral imaging microscopy of macrophage populations in the lung. A) Representative images (20X) of multiplex panel in the lung of uninfected and infected macaques. B) Between-groups comparison of macrophage and EBOV (VP35) markers by positive tissue area. Mann-Whitney Test with Benjamini, Krieger, and Yekutieli correction for multiple comparisons (False Discovery Rate: 5%) was performed (*p<0.05). C) Representative image (20X) of $VP35^{+}CD68^{+}$ double-positive cells in EBOV-infected macaques. n=5 macaques/group.

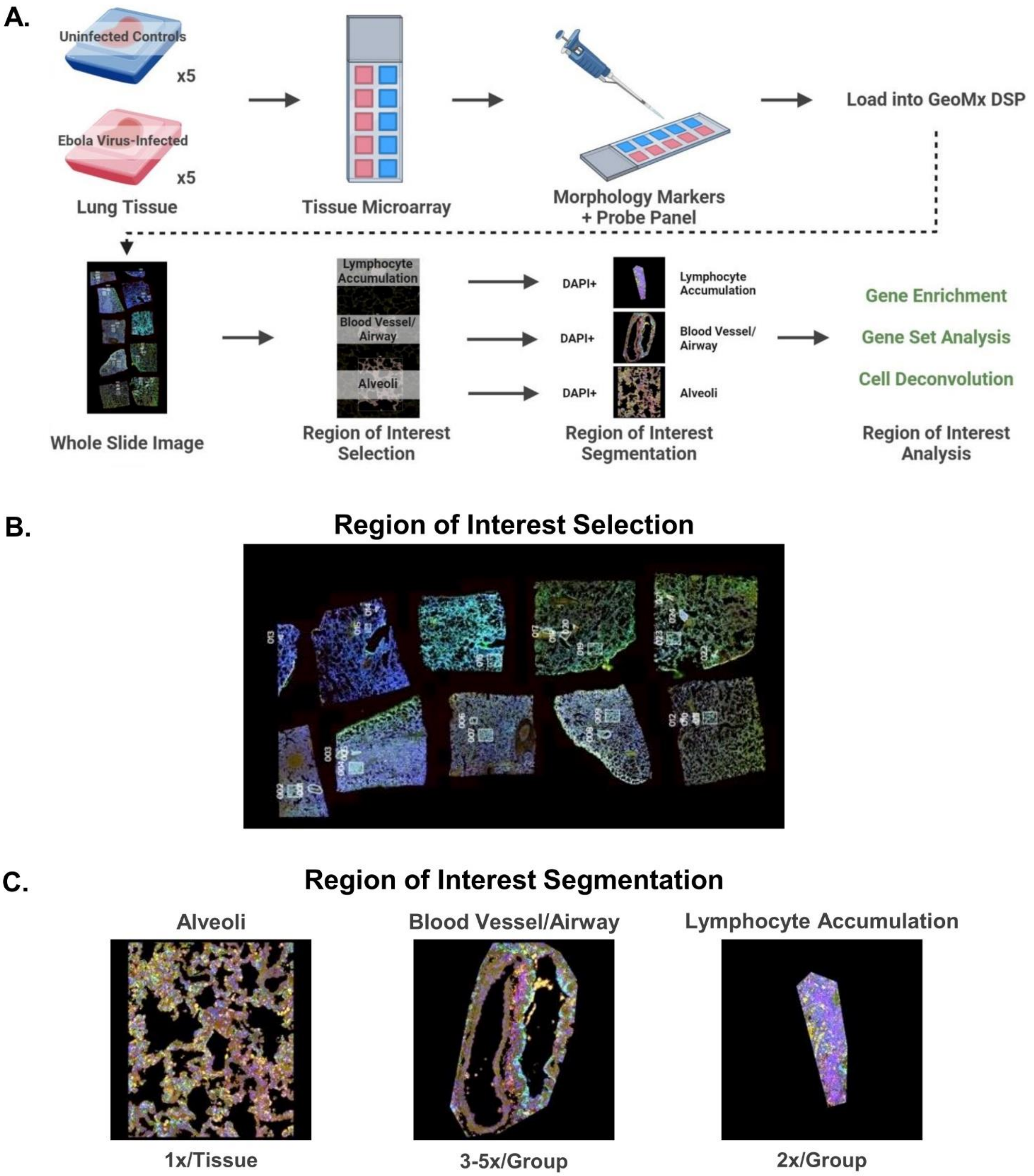

Figure S5. ***In situ*** **protein and whole transcriptome panel analysis of lung tissue from uninfected and EBOV-infected cynomolgus macaques using GeoMx Digital Spatial Profiling technology.** A) Outline of sample preparation, GeoMx DSP procedure, and data analysis. B) Whole slide scan of tissue microarray showing the regions of interest selected for analysis on each tissue. C) Representative images of the region of interest types for GeoMx DSP analysis. Created in Biorender.com.

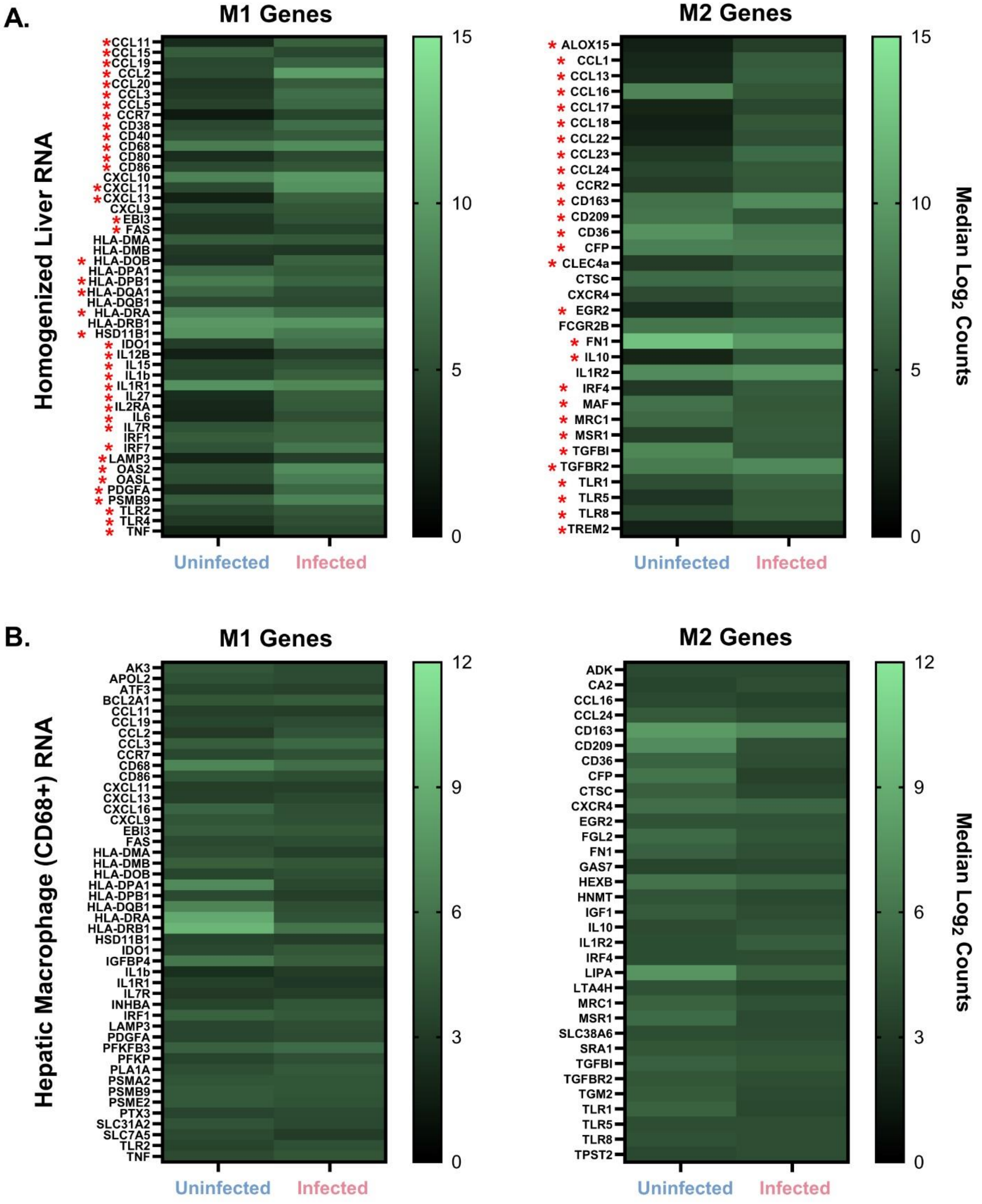

Figure S6. M1- and M2-related gene expression in homogenized liver and hepatic macrophages ($CD68^+$) in uninfected and infected macaques. A) Heatmap of M1- and M2-related gene expression in homogenized liver analyzed by nCounter. B) Heatmap of M1- and M2-related gene expression in hepatic macrophages ($CD68^+$) analyzed by GeoMx DSP. Median expression values (log2 counts) plotted. Mann-Whitney Test with Benjamini, Krieger, and Yekutieli correction for multiple comparisons (False Discovery Rate: 5%) was performed (*p<0.05). Groups: EBOV-infected (n=5) and uninfected macaques (n=5).

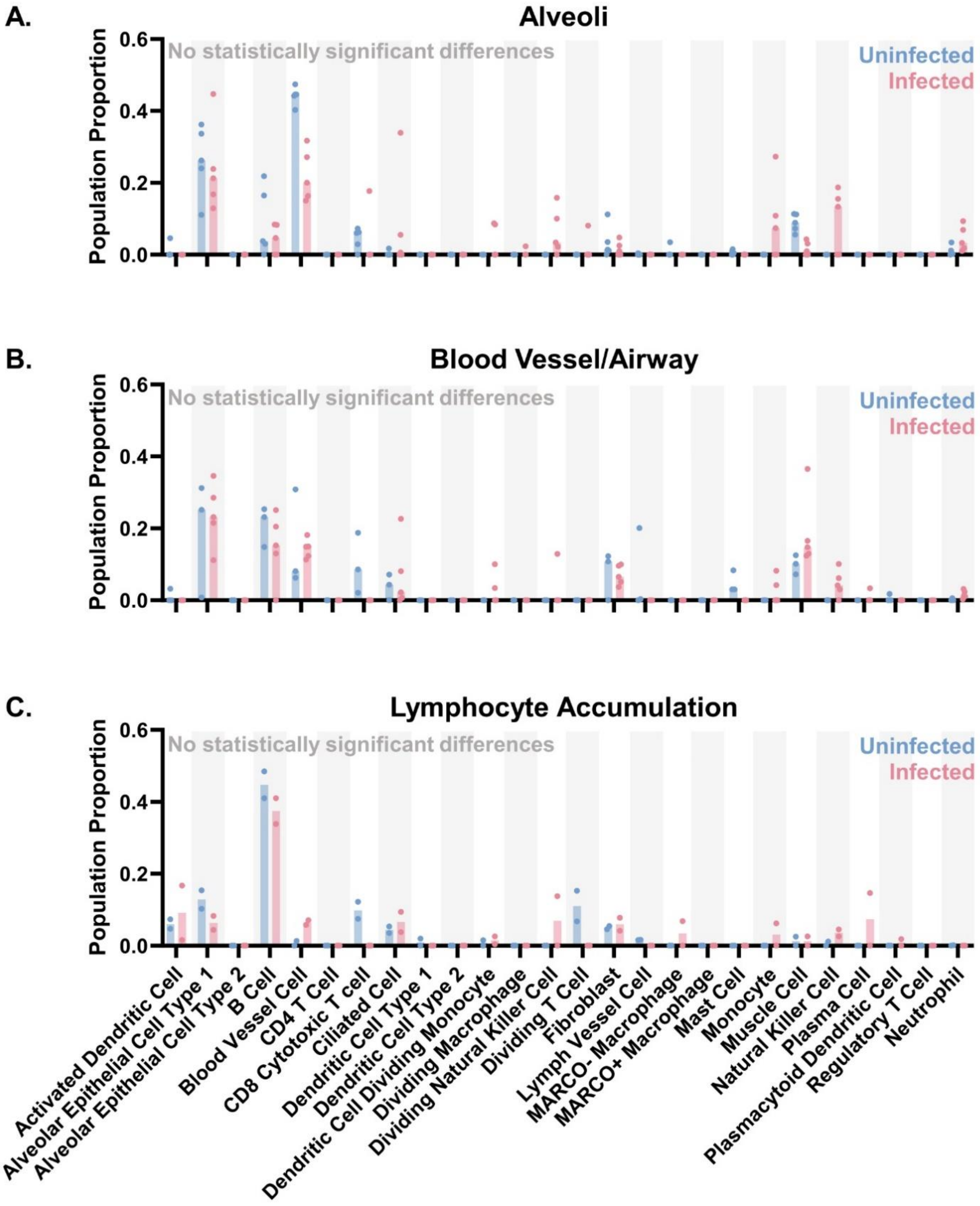

Figure S7. Lung region of interest populations predicted by cell deconvolution in uninfected and infected macaques. Cell deconvolution of the whole transcriptome GeoMx data using a lung cell library was performed for the A) alveolar, B) blood vessel/airway, and C) lymphocyte accumulation regions of interest. Median plotted with data points. Mann-Whitney Test with Benjamini, Krieger, and Yekutieli correction for multiple comparisons (False Discovery Rate: 5%) was performed (no statistically significant differences identified, threshold of $p<0.05$). Group sizes: Alveoli: EBOV-infected (n=5) and uninfected macaques (n=5), Blood Vessel/Airway: EBOV-infected (n=5) and uninfected macaques (n=3), Lymphocyte accumulation: EBOV-infected (n=2) and uninfected macaques (n=2).

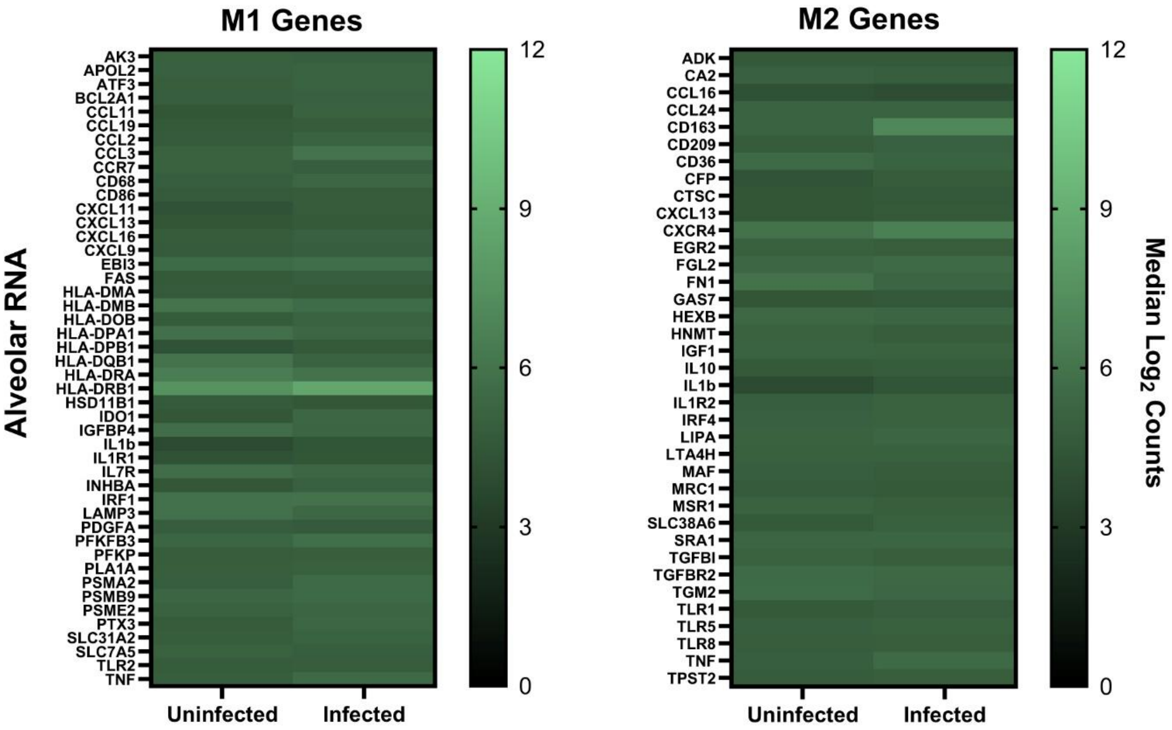

Figure S8. M1- and M2-related gene expression in pulmonary alveoli in uninfected and infected macaques. Heatmaps of M1- and M2-related gene expression in pulmonary alveoli analyzed by GeoMx DSP. Median expression values (log2 counts) plotted. Mann-Whitney Test with Benjamini, Krieger, and Yekutieli correction for multiple comparisons (False Discovery Rate: 5%) was performed (no statistically significant differences identified, threshold of $p<0.05$). Groups: EBOV-infected (n=5) and uninfected macaques (n=5).

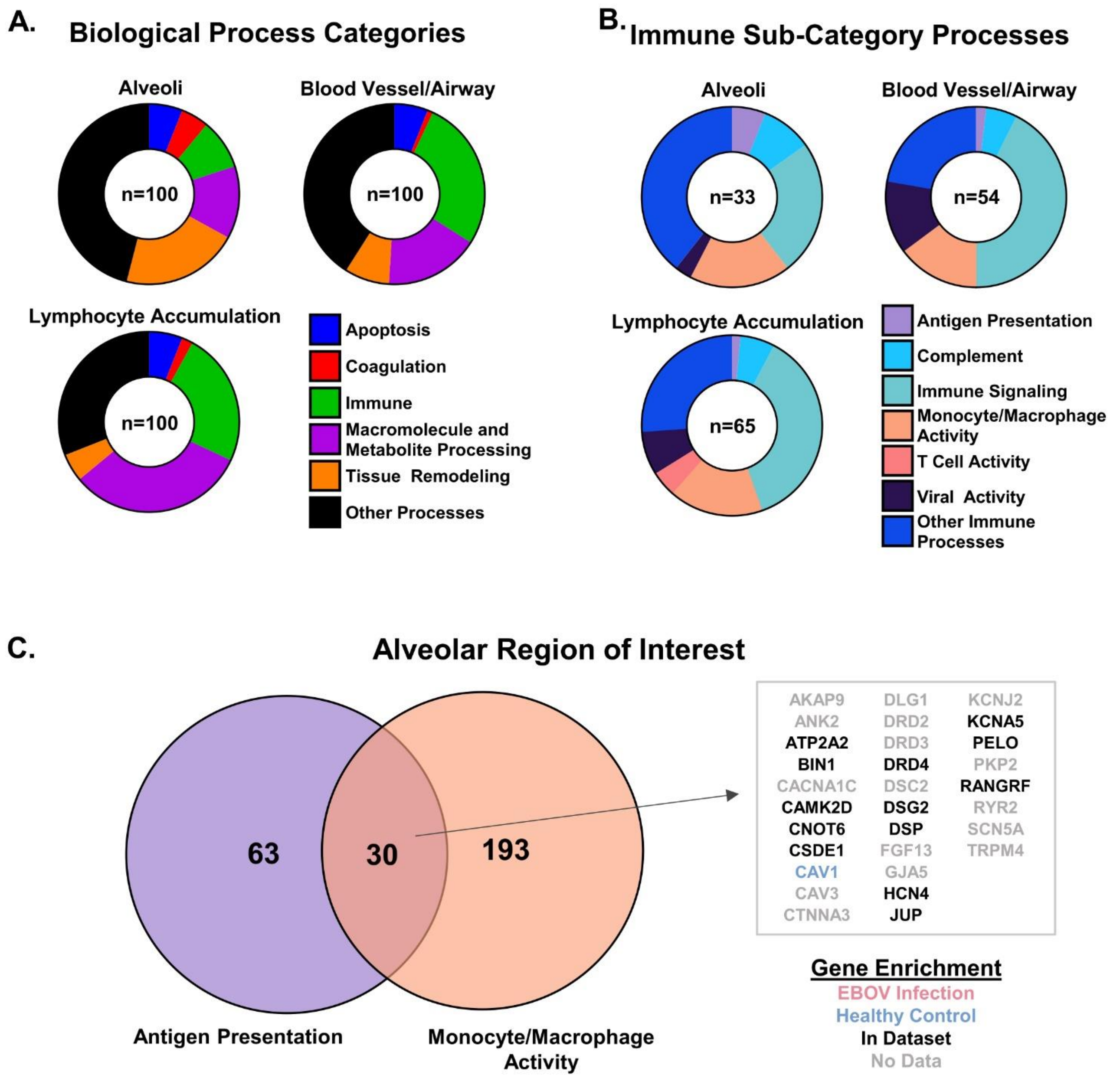

Figure S9. Differential expression of antigen presentation and monocyte/macrophage gene sets in alveolar regions of interest in EBOV-infected macaques. A) Differentially expressed gene set categories by region of interest type identified by Global Test analysis (the numbers in the center of each circle represent the total number of differentially expressed gene sets). B) Differentially expressed gene set sub-categories within the Immune category. Results of Global Test analysis within the Immune category (the numbers in the center of each circle represent the total number of differentially expressed gene sets). C) Differentially expressed genes within the antigen presentation and monocyte/macrophage activity categories (No Data: not measured in GeoMx assay but present in Gene Ontology gene sets). Individual gene color coding was done according to the results of the GeoMx whole transcriptome analysis comparing EBOV-infected macaques (n=5) to uninfected macaques (n=5) (see Figure S10).

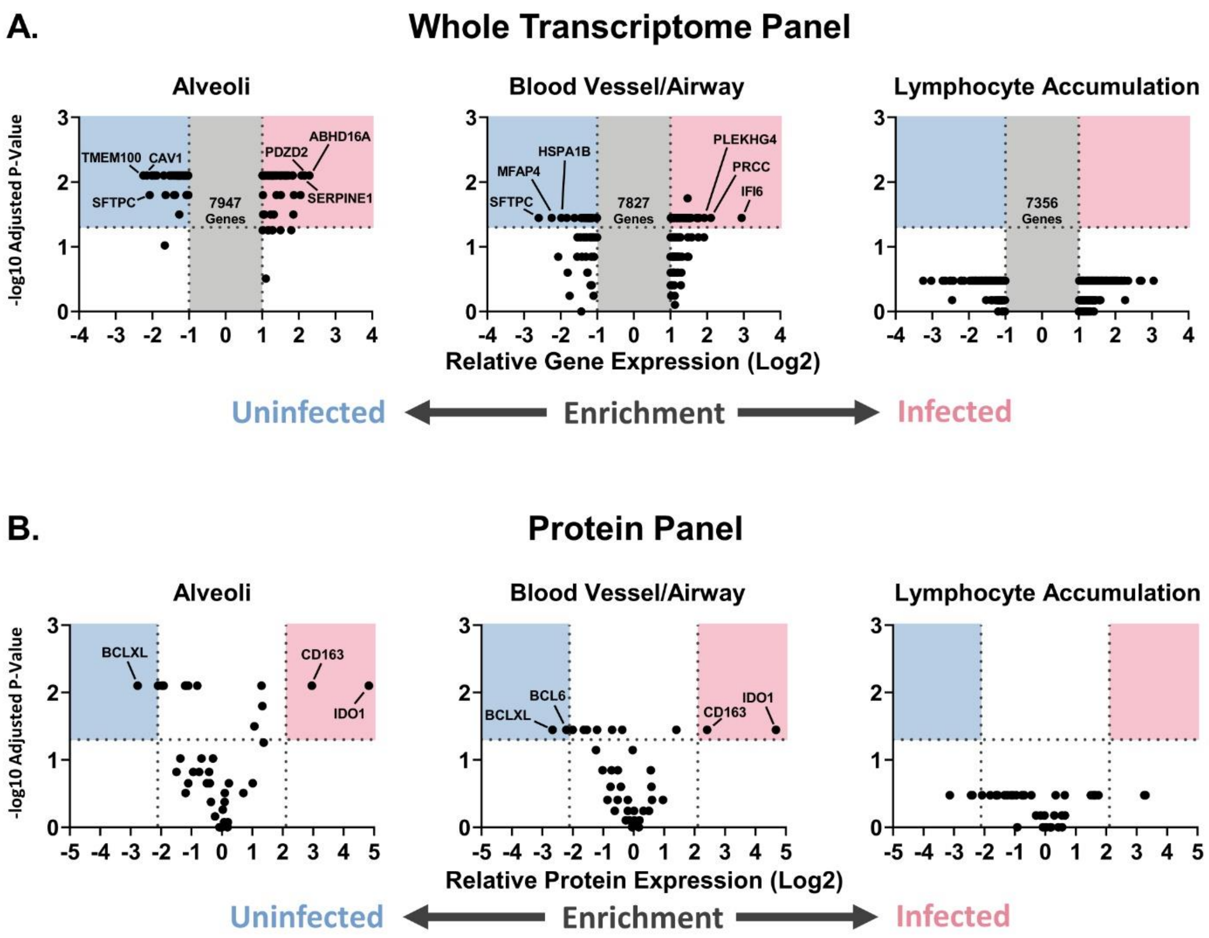

Figure S10. Identification of macrophage- and T cell-related genes/proteins enriched in the lung of EBOV-infected cynomolgus macaques for which there are commercially available therapeutics. A) Volcano plot of genes enriched in uninfected or infected macaques, with the three most enriched genes being labelled in each category, from the whole transcriptome analysis. B) Volcano plot of proteins enriched in uninfected or infected macaques. Mann-Whitney test with Benjamini, Krieger, and Yekutieli correction for multiple comparisons (False Discovery Rate: 5%) ($p<0.05$) on the regions of interest from macaques in the uninfected and infected groups (For the whole transcriptome panel, only genes exceeding the relative expression thresholds were statistically analyzed). Groups: Alveoli: EBOV-infected (n=5) and uninfected macaques (n=5), Blood Vessel/Airway: EBOV-infected (n=5) and uninfected macaques (n=3), Lymphocyte accumulation: EBOV-infected (n=2) and uninfected macaques (n=2).

4 GlobalTest R Script

```
# This code runs a GlobalTest on a dataset.

#Load Necessary R Libraries
library(AnnotationDbi)
library(Biobase)
library(BiocGenerics)
library(IRanges)
library(S4Vectors)
library(org.Hs.eg.db)
library(globaltest)

#Load Gene Data
dataFile <- "Insert tab-delimited text file directory for gene data file location here, in between the quotation marks"
data <- as.matrix(read.table(dataFile, header=TRUE, sep="\t", row.names=1, check.names=FALSE, as.is=TRUE))

#Load Group Definition File
pDataFile <- "Insert tab-delimited text file directory for group information table here, in between the quotation marks"
pData <- read.table(pDataFile, row.names=1, header=TRUE, sep="\t")
all(rownames(pData)==colnames(data))
metadata <- data.frame(labelDescription= c("ROI", "Group", "Macaque"), row.names=c("ROI", "Group", "Macaque"))
pAnnotatedData <- new("AnnotatedDataFrame", data=pData, varMetadata=metadata)


#Load List of Gene Symbol and Entrez ID Match
genes <- "Insert tab-delimited text file directory for gene symbol/Entrez ID key here, in between the quotation marks"
genes <- as.matrix(read.table(genes, header=FALSE, sep="\t", check.names=FALSE, as.is=TRUE))
geneList=as.list(as.integer(genes[,2]))
names(geneList) <- (genes[,1])

#Create Expression Set with Study Data
expressionSet <- ExpressionSet(assayData=data, phenoData=pAnnotatedData)

#Run Global Test on Gene Data
results <- gtGO(Group, expressionSet, probe2entrez = geneList, annotation="org.Hs.eg.db", ontology = "BP", minsize=10, maxsize=100, multtest="BY", permutations = 40000)
resultsFrame <- data.frame (results@result, results@extra)

#Export Gene Data

write.csv(resultsFrame, "Insert csv file directory here for where the data should be saved, in between the quotation marks")
```
